# Validation of Fourier Transform Infrared Spectroscopy for Serotyping of Streptococcus pneumoniae

**DOI:** 10.1128/jcm.00325-22

**Published:** 2022-06-14

**Authors:** I. Passaris, N. Mauder, M. Kostrzewa, I. Burckhardt, S. Zimmermann, N. M. van Sorge, H.-C. Slotved, S. Desmet, P.-J. Ceyssens

**Affiliations:** a Bacterial Diseases Unit, Sciensano, Brussels, Belgium; b Bruker Daltonic GmbH, Bremen, Germany; c Department of Infectious Diseases, Microbiology and Hygiene, University Hospital of Heidelberg, Heidelberg, Germany; d Department of Medical Microbiology and Infection Prevention, UMC Amsterdam, University of Amsterdam, Amsterdam, The Netherlands; e Netherlands Reference Laboratory for Bacterial Meningitis, UMC Amsterdam, University of Amsterdam, Amsterdam, The Netherlands; f Department of Bacteria, Parasites and Fungi, Statens Serum Institut, Copenhagen, Denmark; g National Reference Centre for (invasive) S. pneumoniae, UZ Leuven, Leuven, Belgium; h Department of Microbiology, Immunology and Transplantation, KU Leuven, Leuven, Belgium; Johns Hopkins

**Keywords:** FT-IR spectroscopy, *Streptococcus pneumoniae*, machine learning, pneumococcus, serotyping

## Abstract

Fourier transform infrared (FT-IR) spectroscopy (IR Biotyper; Bruker) allows highly discriminatory fingerprinting of closely related bacterial strains. In this study, FT-IR spectroscopy-based capsular typing of Streptococcus pneumoniae was validated as a rapid, cost-effective, and medium-throughput alternative to the classical phenotypic techniques. A training set of 233 strains was defined, comprising 34 different serotypes and including all 24 vaccine types (VTs) and 10 non-vaccine types (NVTs). The acquired spectra were used to (i) create a dendrogram where strains clustered together according to their serotypes and (ii) train an artificial neural network (ANN) model to predict unknown pneumococcal serotypes. During validation using 153 additional strains, we reached 98.0% accuracy for determining serotypes represented in the training set. Next, the performance of the IR Biotyper was assessed using 124 strains representing 59 non-training set serotypes. In this setting, 42 of 59 serotypes (71.1%) could be accurately categorized as being non-training set serotypes. Furthermore, it was observed that comparability of spectra was affected by the source of the Columbia medium used to grow the pneumococci and that this complicated the robustness and standardization potential of FT-IR spectroscopy. A rigorous laboratory workflow in combination with specific ANN models that account for environmental noise parameters can be applied to overcome this issue in the near future. The IR Biotyper has the potential to be used as a fast, cost-effective, and accurate phenotypic serotyping tool for S. pneumoniae.

## INTRODUCTION

Streptococcus pneumoniae is a Gram-positive opportunistic pathogen that can cause a wide variety of diseases, ranging from more mild infections, such as sinusitis, otitis media, and conjunctivitis, to more severe diseases, such as community-acquired pneumonia (CAP), bacteremia, and meningitis (1–5). The World Health Organization (WHO) estimated that S. pneumoniae infections still killed close to 300,000 children under 5 years old worldwide in 2015 (6). Moreover, in 2016, and thus before the COVID-19 pandemic, S. pneumoniae was the leading cause of pneumonia-induced mortality worldwide, accounting for more deaths than all other etiologies causing lower respiratory tract infections combined (7).

The cells of most S. pneumoniae strains are surrounded by a polysaccharide capsule that protrudes into the extracellular space (8, 9). The capsule has been shown to be an important virulence determinant and has been linked to different roles in the infection process (10), including (i) inhibition of both the classic and alternative complement pathways, which reduces opsonophagocytic killing (11, 12), and (ii) promotion of nasopharyngeal colonization (13). Currently, around 100 different chemical capsular structures have been described and categorized into serotypes (8, 14). However, only a limited number of pneumococcal serotypes cause the large majority of disease around the world, and different vaccines have been developed to target the ones dominant in invasive diseases (15, 16). The pneumococcal polysaccharide vaccine (PPSV23) has been on the market since the 1980s and protects against 23 serotypes but with a moderate long-term immunity (16–18). More importantly, this vaccine does not protect infants below the age of two, which is the age group with the highest incidence of severe disease and the highest level of carriage. The protein conjugate vaccines (PCVs) were first implemented in the U.S. childhood vaccination programs in the early 2000s because of their projected efficacy in infants, as well as their promising potential in providing long-lasting immunity (19–22). The current PCVs protect against 10 (PCV10) or 13 (PCV13) different serotypes, but in the meantime, higher valency PCVs (PCV15 and PCV20) have been licensed for adults and are (or soon will be) available (23–27). The surveillance of circulating serotypes in the population is therefore a critical epidemiological tool for assessing the impact of vaccination on the distribution of serotypes (28, 29).

Historically, the identification and classification of pneumococcal serotypes is based on reaction with specific rabbit polyclonal antisera (8). Visual inspection using light microscopy and the observation of “swollen” capsules (when antibodies have reacted with and bound a specific capsule) defines a positive reaction. This particular serotyping method is known as the quellung reaction and has been used for over 100 years to provide information on the chemical structure of the capsule (30, 31). More recently, the quellung reaction has been complemented by other phenotypic techniques, of which latex agglutination has been the more widely adopted because of its speed of use and simplicity compared to the quellung reaction (32). Although these phenotypic methods are still used routinely in national reference centers as the gold standard for serotyping S. pneumoniae isolates, there are disadvantages linked to them (33). First, the interpretation can be subjective and requires well-trained and experienced technicians (34–36). Second, performing these techniques is time consuming and labor intensive (typically 5 to 30 min per strain is required, depending on the specific serotype) and they are not well suited for high-throughput serotyping. Finally, the required typing antisera are expensive. Overall, this has limited the use of these phenotypic methods as a routine method to a limited number of clinical laboratories (mostly national reference centers) (37).

Over the years, various molecular alternatives have been developed, mostly using the genetic information of a particular pneumococcal strain and translating this into a serotype (33). First of all, *cpsB* “sequetyping” has been proposed as a simple genetic approach (only one standard PCR is required) based on the genetic diversity between *cpsB* alleles of different serotypes (38). Albeit easy to use, the technique suffers from relatively low sensitivity and specificity (around 85%), is often limited to identification to the serogroup (SG) level, and only works for a subset of serotypes/groups (33, 38). Multiplex real-time (RT)-PCR overcomes some of these limitations, targeting up to 34 serotypes and 13 small serogroups in 12 reactions and achieving high sensitivity (39). The high-throughput nature of multiplex RT-PCR is an advantage compared to whole-genome sequencing (WGS), the most recent genetic approach that has gained momentum in the last decade (34, 35). Different pneumococcal serotype prediction algorithms have been developed that perform with high accuracies, such as PneumoCat (35) (>99% sensitivity), SeroBA (34) (98% sensitivity), and SeroCall (40) (100% sensitivity for major serotypes and up to 86% for minor serotypes). However, to date, WGS remains a rather slow procedure, and this approach, similar to *cpsB* sequetyping and RT-PCR, does not measure the chemical composition of the capsule as such. Overall, capsule gene sequencing is able to accurately detect genetic changes like single-nucleotide polymorphisms (SNPs) or recombination events, but the impact of these changes on the capsule structure or expression cannot be deduced.

A promising technique that does provide information on the capsule structure itself is Fourier transform infrared (FT-IR) spectroscopy (41). With FT-IR spectroscopy, biological material is exposed to infrared light and an absorption spectrum is generated (42). The region of interest for capsular typing (fingerprinting region) is located between wavenumbers 800 and 1,300 cm^−1^, where the carbohydrates are predominantly absorbing (C-O stretching and O-H bending). This technique has shown potential in serotyping (or serogrouping) different bacteria, such as Salmonella (43–45), Klebsiella (46, 47), Enterobacter (47, 48), Acinetobacter (47), and Pseudomonas (47), and has the advantage of being easy to perform, cost effective and medium throughput. Furthermore, FT-IR spectroscopy has already been described in the literature as a promising serotyping technique for S. pneumoniae, but it is only quite recently that Burckhardt et al. (37) have provided a first comprehensive validation of the technique.

In this study, FT-IR spectroscopy was further evaluated as a serotyping technique for S. pneumoniae. An accurate serotyping workflow was developed through analysis of an in-house-constructed training set of 233 international reference strains (representing 34 serotypes) in combination with a validation set of 277 S. pneumoniae strains (representing 93 different serotypes), using dimensionality reduction analysis, hierarchical cluster analysis (HCA), and a machine-learning algorithm. The robustness of the method was evaluated, and possible bottlenecks for the standardization and wide implementation of FT-IR spectroscopy for pneumococcal serotyping were investigated.

## MATERIALS AND METHODS

### Strains and growth conditions.

S. pneumoniae reference and validation strains were either part of our in-house collection (49, 50) or obtained from the reference centers for pneumococcal diseases of Belgium, The Netherlands, and Denmark (Table S1 in the supplemental material). Isolates from Belgium and The Netherlands were serotyped using the quellung reaction, while isolates from Denmark were serotyped either by the quellung reaction alone or by the pneumotest latex kit (SSI Diagnostica, Denmark) combined with the quellung reaction. For the quellung reaction, type-specific rabbit pneumococcal antisera (SSI Diagnostica, Denmark) were used, and the method was performed as described previously (36, 51, 52). Isolates from Belgium and Denmark were stored in brain heart infusion (BHI) broth with 10% glycerol at −80°C, while strains from The Netherlands were received and stored in peptone broth with 8% glycerol at −80°C. Strains at Sciensano were incubated directly from a −80°C stock cryotube on Columbia agar base medium plus 5% sheep blood (CB + 5% SB) (Oxoid) and grown for 24 h at 36°C in 5% CO_2_ (Binder C170 incubator; VWR) before being measured.

There were some small differences in the incubation protocols of the other sites (Bruker and University Hospital of Heidelberg). At Bruker, isolates were incubated from a −80°C Microbank (Thermo Fisher Scientific) on CB + 5% SB (Becton Dickinson [BD]) at 35°C to 37°C with a 2.5-L CO_2_ gen pack (Thermo Fisher Scientific). The next day, isolates were subcultured on fresh CB medium and incubated for 24 h under the same conditions, after which FT-IR spectroscopy measurements were performed. At the University Hospital of Heidelberg (UHH), strains were incubated on CB + 5% SB (BD) for 24 h at 36°C in 5% CO_2_ (53) and subsequently subcultured for another 24 h on fresh CB, after which FT-IR spectroscopy measurements were performed.

### FT-IR spectroscopy.

Pneumococcal strains were prepared for FT-IR spectroscopy using the direct smear method. Briefly, a 1-μL plastic loop was used to carefully remove the biomass from the plate and apply it evenly to a silicon plate containing 96 spots (Bruker, Germany). Apart from the pneumococcal strains, two E. coli reference strains (IRTS 1 and IRTS2; Bruker) were applied to the silicon plate to validate the run. After the samples were dried, the silicon plate was placed under a UV lamp (25 W; Philips) at a distance of 5 cm for 10 min to inactivate the bacterial cells. Finally, the plate was inserted into the IR Biotyper (Bruker, Germany) and the measurements were started.

### Analysis of FT-IR spectroscopy spectra. (i) HCA: the dendrogram.

Analysis of the spectra was partly performed with the IR Biotyper software (version 3.0) and partly using third party software (Excel, RapidMiner, and PAST). First, average spectra were created from all spectra of each individual strain using the IR Biotyper software and, in the case of the training set strains, further labeled with their respective serotypes. Next, all individual and average spectra were exported as a spreadsheet in XLS format, creating a large data set containing 521 data points (between wavenumbers 800 and 1,300 cm^−1^) for each spectrum. All individual spectra were subsequently used to build a principal-component analysis (PCA) model with the RapidMiner Studio 9.8 software using a variance threshold of 0.999, after which the freshly built model was applied to all average spectra. The resulting simplified data set now contained all average spectra of the 233 strains with 86 principal components (PCs) and was subjected to linear discriminant analysis (LDA) using the open-source PAST4.03 software (54). As a final analysis step, hierarchical cluster analysis (HCA) was applied to all 233 strains and their respective 34 linear discriminants using Ward’s algorithm as the linkage type, generating a large dendrogram containing all 233 training set strains. For the analysis of the validation set strains, the analysis procedure was the same, except that these strains, labeled “SAMPLE” of unknown serotype (“?”), did not contribute to building the PCA model and did not influence the supervised LDA model, as they were unlabeled. The validation set strains appear after HCA in the dendrogram in the cluster of strains most closely resembling them.

### (ii) Artificial neural network model: the PneumoClassifier.

The IR Biotyper software offers the possibility to use machine-learning algorithms on the measured spectra. Two PneumoClassifiers were developed (version 1.0 and version 2.1) and varied in the numbers of spectra used for training and/or the numbers of training cycles. The accuracy of the classifiers was determined in a stratified 4-fold cross-validation test with a confusion matrix as the output.

Finally, validation strains were analyzed in technical pentaplicates and the resulting average spectrum was used by PneumoClassifier version 2.1 to predict the serotype. A green-colored serotyping output (outlier value of <1.4) signified that the result was within the boundaries of the training set and could be trusted, while an orange-colored serotyping output (outlier value between 1.4 and 2.0) signified that the result was at the boundaries of the training set and should be interpreted with care. When the result was colored red (outlier value of >2), the result was outside the boundaries of the training set and thus could not be trusted.

### Whole-genome sequencing (WGS).

Overnight S. pneumoniae cultures of all serogroup 6 (SG6) training set strains (Table S2) were scraped from the plates and dissolved in BHI broth (BD), and DNA was extracted using an MgC Bacterial DNA Kit (Atrida, Amersfoort, The Netherlands), following the manufacturer’s instructions. Sequencing libraries were prepared using the Illumina Nextera XT DNA sample preparation kit according to the manufacturer’s instructions (Illumina, San Diego, CA, USA), and DNA sequencing was performed on an Illumina MiSeq instrument (MiSeq version 3 chemistry). Trimming, clipping, filtering out non-confident bases, correcting sequencing errors, reducing redundant reads, and merging overlapping paired reads were performed using fqCleanER version 3.01 (55–57). All FASTQ files were uploaded to Pathogenwatch (https://pathogen.watch/), which uses the SeroBA (34) algorithm to predict the pneumococcal serotype. In addition to this, a more detailed analysis was performed by looking into the *wciP* and *wciN* genes, which are known to be the genetic determinants of the different SG6 capsule structures (58–61). In short, FASTQ reads were imported into CLC Genomics Workbench 21.0.4 (Qiagen) and mapped in parallel to the *cps* clusters of both an SG6 subtype B (6B) (NCBI accession number CR931639) and a 6C (NCBI accession number EF538714) reference strain. Strains with the *wciP* (584A) single-nucleotide polymorphism (SNP) were either 6B or 6D, while strains with the *wciP* (584G) SNP were either 6A or 6C. Information on which *wciN* variant is present (*wciNα* in 6A and 6B and *wciNβ* in 6C and 6D) in these strains completes the subtyping process.

### Data availability.

All sequences have been made publicly available in the NCBI Sequence Read Archive and can be retrieved using the accession number PRJNA836581.

## RESULTS

### Construction of FT-IR spectroscopy training set for S. pneumoniae serotypes.

**FIG 1:**
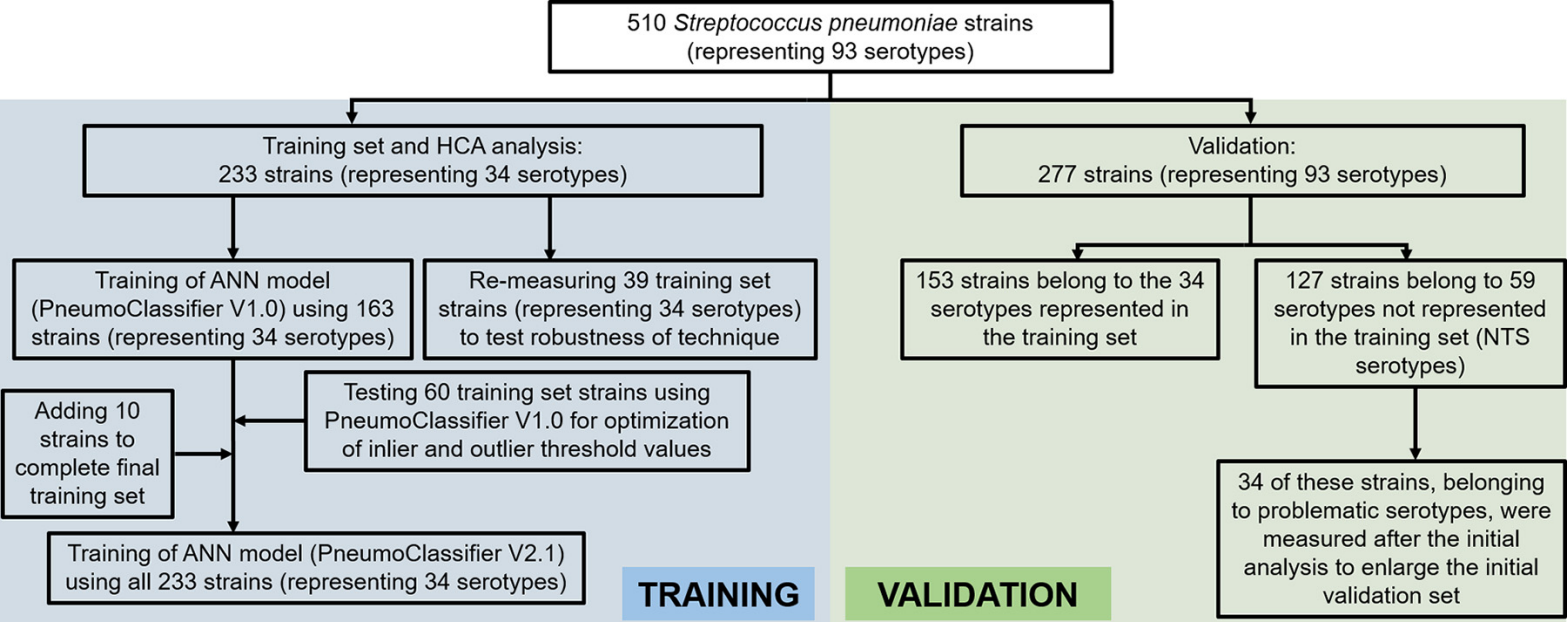
Schematic overview of the S. pneumoniae strains measured with FT-IR spectroscopy and their purpose throughout the study.

A collection of S. pneumoniae isolates from Belgium (173 strains) and The Netherlands (60 strains) from patients with invasive pneumococcal disease (IPD) (178 strains) or noninvasive pneumococcal disease (NIPD) (55 strains) was analyzed to create a robust database (Fig. 1 and Table S1). These 233 strains represented 34 different serotypes (24 VTs and 10 NVTs), with each serotype being represented by at least 4 different strains (Table 1). All strains were serotyped using the quellung reaction (and/or the latex agglutination test) and were representative of the circulating serotypes causing IPD in Belgium/Europe (51, 62, 63).

**TABLE 1:** Serotypes represented in the training set and the respective numbers of strains used

Serotype (*n* = 34)	No. of strains in training set (*n* = 233)
1	4
2	5
3	9
4	5
5	4
6A	8
6B	6
6C	11
7F	7
8	5
9V	7
9N	6
10A	5
11A	9
12F	5
14	10
15A	9
15B	10
16F	6
17F	6
18C	5
19A	9
19F	11
20	5
22F	6
23A	7
23B	10
23F	5
24A	4
24B	5
24F	8
33F	6
35B	10
38	5

**FIG 2:**
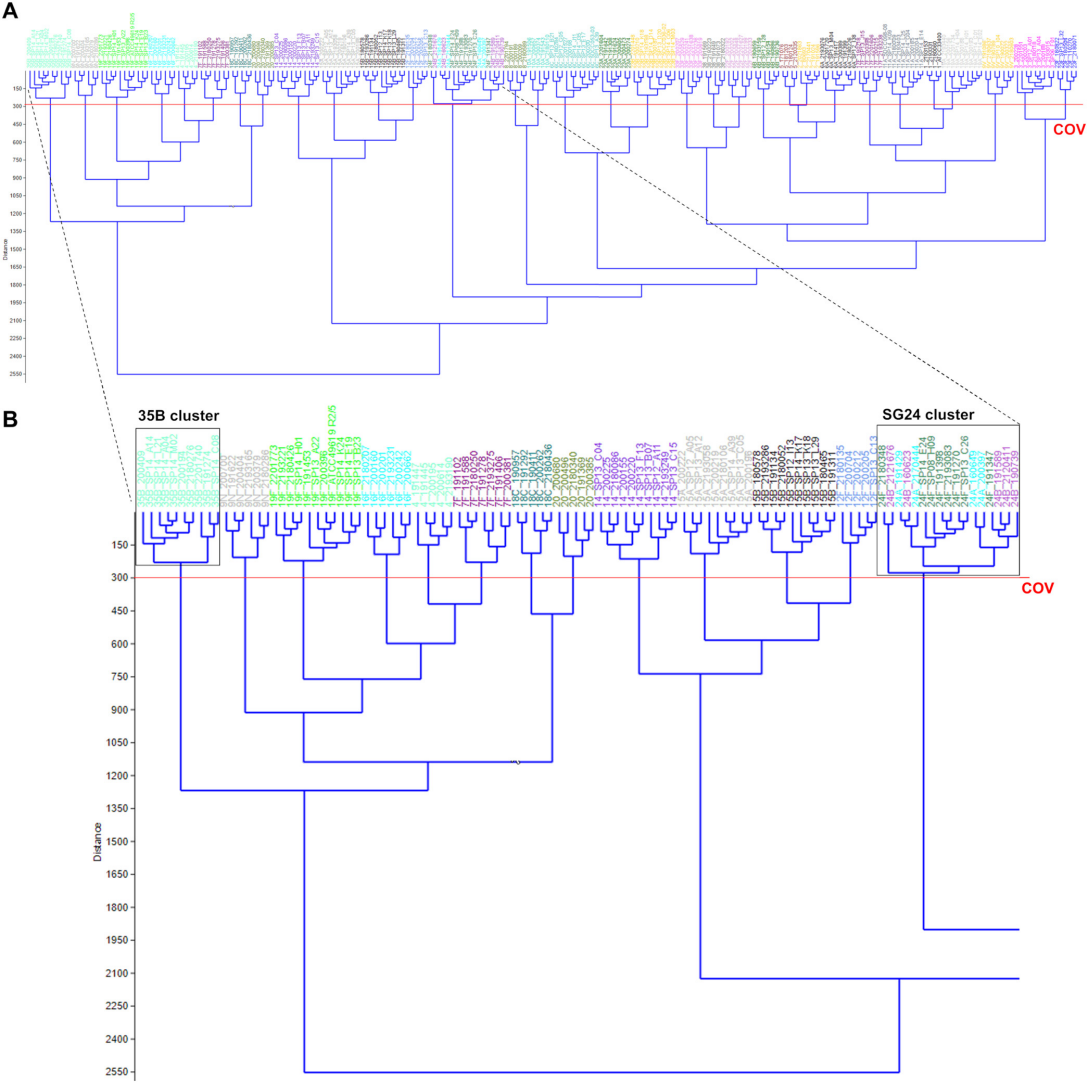
(A) Dendrogram visualizing the 233 pneumococcal training set strains representing 34 serotypes. The red line depicts the cutoff value (COV), separating strains from different serotypes into distinct clusters but at the same time grouping strains from the same serotype in one cluster. The linkage type used for the dendrogram was Euclidean distance with Ward’s algorithm. (B) Zoomed-in image of the outer left part of the dendrogram shown in panel A (dashed lines). Different clusters grouping strains from the same serotypes can be observed (e.g., 35B cluster). The SG24 cluster is shown as a clearly distinct cluster, but within this cluster, the different subtypes (24A, 24B, and 24F) are not accurately separated.

All 233 pneumococcal strains were measured using FT-IR spectroscopy on three independent occasions, yielding a minimum of 12 spectra per strain. This large and complex data set was analyzed using a combination of dimensionality reduction techniques (PCA, grouped by isolate, and LDA, grouped by serotype) and hierarchical cluster analysis (Euclidean distance with Ward’s algorithm linkage type). The exact workflow is described in detail in Materials and Methods. The chosen output for this analysis procedure was a dendrogram (Fig. 2A), a tree-shaped figure with one stem splitting into two branches, eventually ending in 233 branch tips, with all strains represented and forming clusters. The vertical scale represents the distance between strains, so that the more similar they are, the later they split off, eventually ending up in clusters. The cutoff value (COV) for the dendrogram was chosen manually, with the goals being (i) to separate as many of the different serotypes as possible into different clusters and (ii) to group as many individual strains of the same serotype as possible within one cluster.

When interpreting the dendrogram for the strains in our training set strains, it is clear that almost all isolates of the same serotype cluster together and different serotypes form distinct clusters (a detailed view is presented in Fig. 2B). The exceptions to the rule are the strains from SG24 (subtypes 24A, 24B, and 24F) that form one distinct serogroup cluster but cannot be accurately separated into their respective subtype clusters (Fig. 2B). Together, these results are highly encouraging and show that FT-IR spectroscopy can accurately separate 31 different pneumococcal serotypes (encompassing all VTs) and group 24A, 24B, and 24F strains at the serogroup level.

### Training an ANN model to classify pneumococcal serotypes.

**FIG 3:**
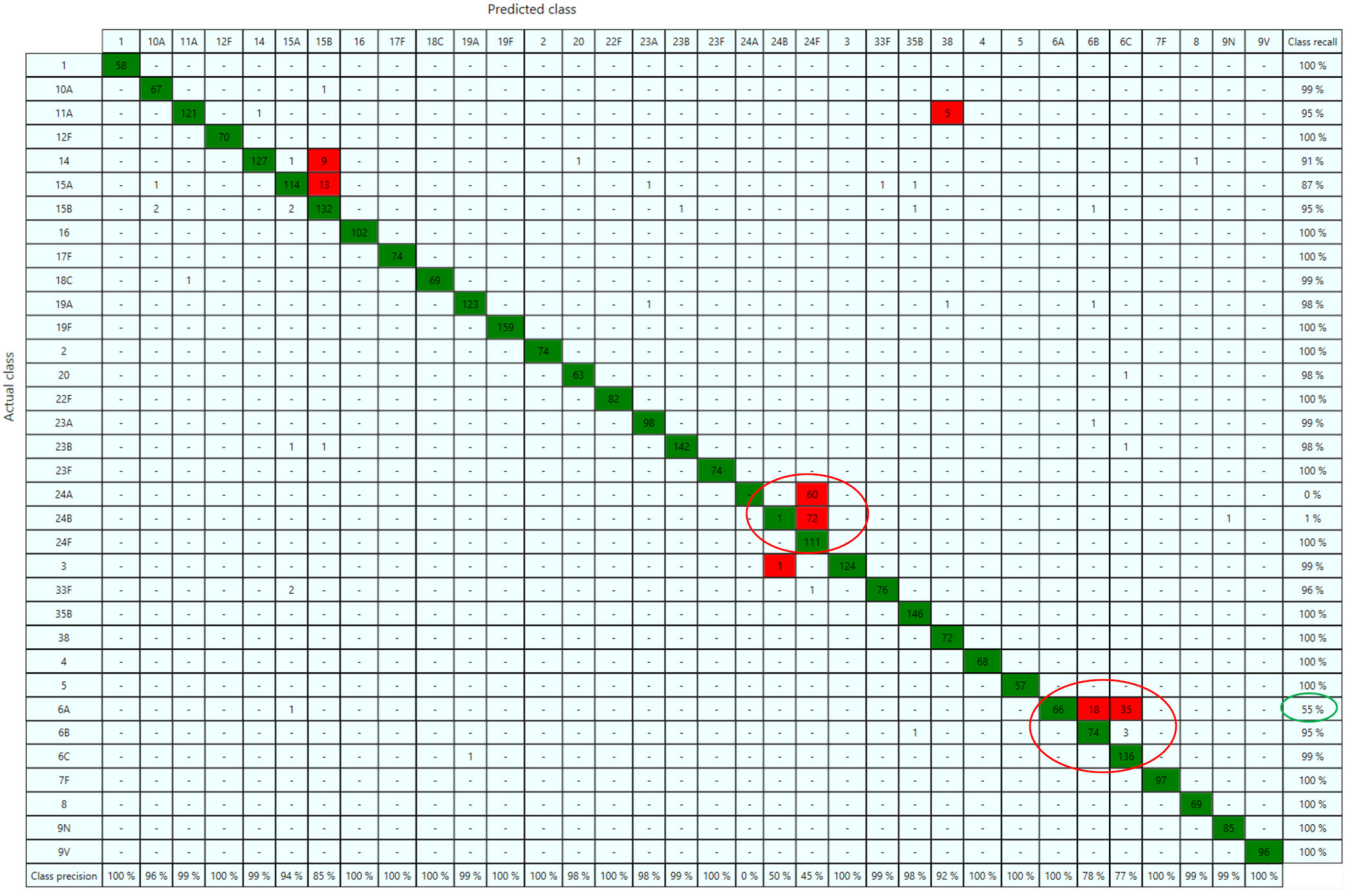
Confusion matrix showing the output of the trained PneumoClassifier version 2.1 (500 training cycles). The highlighted SG24 and SG6 subtypes in the red-encircled areas are spectra that were often misclassified within the serogroup and led to low sensitivities for these serogroups. The green-encircled value is the sensitivity for predicting 6A spectra before the classifier was retrained with the corrected SG6 serotypes.

Using the IR Biotyper (version 3.0) software, an artificial neural network (ANN) model was trained using all individual training set spectra. First, an ANN model was built to use the first 20 principal components (PCs) on a subset of the data set (2,246 spectra; PneumoClassifier version 1.0) and trained for 200 cycles. Using this classifier, 60 training set strains that were not yet incorporated in the model (Fig. 1 and Table S1) were utilized as mock serotyping strains to tweak the inlier and outlier thresholds of the model. It was found that increasing the inlier threshold from 1.0 (default setting) to 1.4 provided a more realistic result in confidently typing training set strains. Next, the final ANN model (PneumoClassifier version 2.1) was created with the full training set (3,276 spectra) and trained for 500 cycles. The PneumoClassifier had a total accuracy of 92% in correctly predicting the serotypes of strains that were in the training set. A confusion matrix was obtained as visual output to get a detailed view of the classifier’s performance for every serotype (Fig. 3). The large majority of errors was found within SG24 (and to a lesser extent within SG6), thus encountering a similar problem as with the dendrogram output. Because SG6 is an important serogroup consisting of both vaccine (6A and 6B) and nonvaccine (6C and 6D) types, all SG6 training set strains were subjected to WGS and the SeroBA algorithm was used for serotype prediction. This resulted in three strains for which a different serotype was predicted by WGS, and after repeating the quellung reaction on these strains, one strain was found to be incorrectly categorized as 6A instead of 6C (Table S2). When this error was corrected and the classifier retrained, the sensitivity for predicting 6A spectra increased from 55% to 85%, thus yielding satisfying accuracies for all SG6 subtypes.

### Validation of the serotyping workflow.

**FIG 4:**
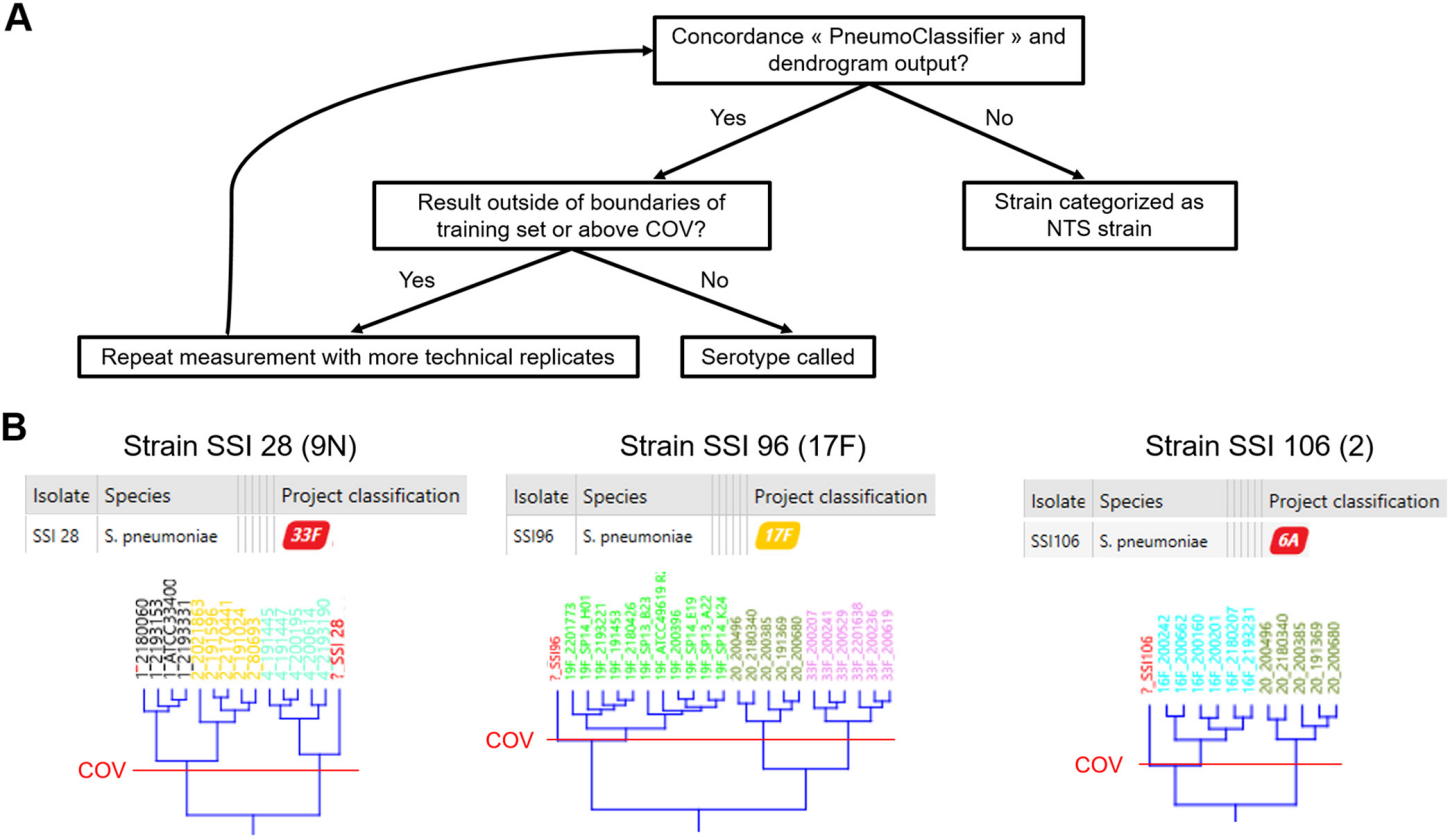
(A) Schematic overview of the serotyping workflow adopted throughout the study. (B) Serotyping output for three validation set strains that were mistyped as NTS strains using the serotyping workflow shown in panel A. Top, the tables show the output of the PneumoClassifier version 2.1; bottom, the relevant part of the dendrogram for each validation set strain is shown. The red lines depict the cutoff value (COV).

To validate the FT-IR spectroscopy approach as an S. pneumoniae serotyping method, a serotyping workflow was established to analyze pneumococcal strains that were not part of the training set (Fig. 4A), as follows. (i) The serotype of an analyzed strain was called when the serotypes obtained via the dendrogram and the PneumoClassifier were concordant and the result was within both the dendrogram cluster demarcated by the COV and the training set of the PneumoClassifier (green or orange code). (ii) A strain was classified as a non-training set serotype (NTS) when the serotypes obtained via the dendrogram and the PneumoClassifier were discordant. (iii) The measurement was repeated with more technical replicates when the serotypes obtained via the dendrogram and the PneumoClassifier were concordant but the result was outside the dendrogram cluster demarcated by the COV or outside the training set of the PneumoClassifier. If there was still any doubt after this, the strain was classified as an NTS serotype.

Using this workflow, a validation set of 277 strains belonging to 93 different serotypes and different from the strains used to train the model were measured with FT-IR spectroscopy (Fig. 1 and Table S1). A total of 153 strains belonged to the 34 serotypes included in the training set, and all of these serotypes were challenged at least once (Table S3). In total, 150 of 153 strains were found to be correctly serotyped (or grouped, in the case of SG24), yielding an accuracy of 98.0% in predicting serotypes included in the training set. Only three strains, belonging to three different serotypes (17F, 9V, and 2), were mistyped as NTS serotypes (Fig. 4B).

Furthermore, a set of 90 validation strains belonging to 59 NTS serotypes (Table S4) were measured initially and analyzed using the serotyping workflow. For the majority of NTS serotypes, one strain was tested. Only serotypes 7D, 10D, 10E, 11E, and 33E could not be tested, as they were lacking in the collections of the reference centers of Belgium, The Netherlands, Denmark, and Germany. Applying the serotyping workflow to the NTS validation strains resulted in 42 of 59 serotypes being correctly classified as NTS serotypes, thus attaining an accuracy of 71.2% in correctly predicting NTS serotypes. The NTS serotypes that were confounded for training set serotypes are listed in Table 2. Of the 17 problematic serotypes, 14 serotypes were only represented by one validation strain, and an extra set of validation strains (*n* = 34, making a total of 124 NTS validation strains) was acquired for 12 of these problematic serotypes (7A, 9L, 9A, 11D, 12B, 15F, 18A, 19B, 29, 33A, 39, and 40). After measuring and analyzing this extra set of strains, all 12 serotypes were confirmed to be confounded with training set serotypes.

**TABLE 2:** Summary of the 17 NTS serotypes that were mistyped as training set serotypes using FT-IR spectroscopy

Serotype from FT-IR spectroscopy^*a*^	NTS serotype from quellung reaction^*b*^	Differences in chemical structures of capsule^*c*^
6C	6D	Difference in positions of glycosidic linkages between adjacent sugar moieties
7F	7A	7F harbors an extra branching sugar
9N	9L	One sugar difference in sugar backbone (glucose vs galactose)
9V	9A	Different amounts of acetylation
10A	39	Difference in acetylation and positions of glycosidic linkages
11A	11D	11D is part 11A and part different sugar backbone (glucose vs *N*-acetylglucosamine) and different acetylation amt
12F	12B	No structural information available on 12B
15B	15C	15B contains acetylated sugar moiety
18C	18A	Difference in acetylation and one sugar moiety (glucose vs *N*-acetylglucosamine)
19A	19B	19B contains an extra sugar in its backbone and additional branching sugars
33F	33A and 33B	33A and 33B differ in amounts of acetylation and 33B contains different sugars in its backbone
15A	15F	Difference in side chains (glycerol vs choline) and acetylation
24	40	No structural information available on 40
35B	29 and 35D	Different sugars in backbones of 35B and 29 and difference in acetylation between 35B and 35D
38	25A	No structural information available on 25A

a Vaccine types (VTs) are shaded in gray.

b NTS, non-training set.

c The main chemical structural differences of the capsules of these serotypes are listed.

### Robustness of the FT-IR spectroscopy technique for pneumococcal serotyping.

FT-IR spectroscopy is a highly sensitive technique, picking up very small chemical differences. This is a big advantage when serotyping strains with very similar capsule structures but can also be potentially troublesome when environmental conditions change (e.g., type of medium and composition, temperature, humidity, and incubation time), sometimes in ways that are beyond the control of the operator, and cause alterations in biomass composition.

To test the robustness of the technique, two approaches were adopted. First, 39 training set strains (with every training set serotype represented at least once) were remeasured 1 year after they had been initially measured to construct the training set (Table S1). Of all the retested strains, 22 were measured in technical pentaplicates and 17 in technical triplicates. Of these 39 strains, 36 strains were serotyped correctly, while 3 strains were incorrectly categorized as NTS serotypes, indicating that a degree of variation (of thus-far-unknown origin) had emerged that could lead to mistyping. The three strains belonged to three different serotypes (23A, 23B, and 6A) and were measured in technical triplicates, suggesting that measuring fewer technical replicates could lead to more mistyping. To evaluate this, the three problematic strains were measured once more in technical pentaplicates, and all were then found to be serotyped correctly.

**FIG 5:**
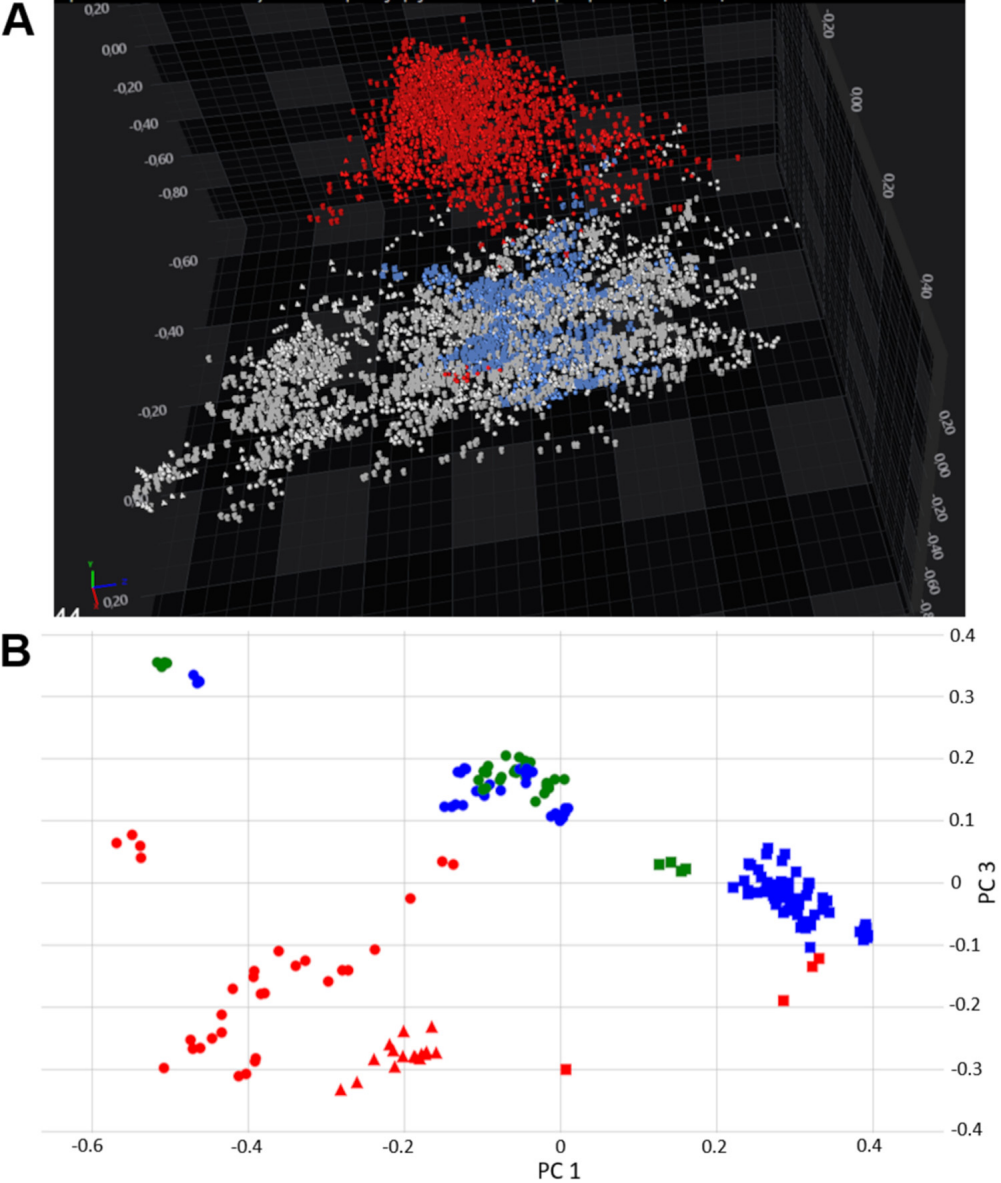
(A) Three-dimensional PCA plot constructed using around 6,000 spectra acquired from three different locations (Sciensano, Brussels; Bruker, Bremen; and UHH, Heidelberg). Data points are colored according to the location where they were measured: red data points at Sciensano, blue data points at UHH, and gray data points at Bruker. The spectra acquired at Sciensano cluster together strongly but far away from the spectra acquired at Bremen and Heidelberg. Together, PC1, PC2, and PC3 account for 60% of the total variation. (B) Two-dimensional PCA plot (PC1, 46.2% of the total variance explained, and PC3, 15.9% of the total variance explained) depicting 184 spectra from strains (representing 9 different serotypes) that were measured on CB + 5% SB from three different suppliers (Oxoid, BD, and bioMérieux). The spectra acquired on Oxoid medium (red data points) cluster away from the spectra acquired on BD (blue data points) and bioMérieux (green data points) media, regardless of the location at which the spectra were measured. Data points with different shapes depict different locations: triangles correspond to Sciensano spectra, squares to UHH spectra, and dots to Bruker spectra.

A second approach to testing the robustness of the technique was to compare spectra acquired at Sciensano (Brussels, Belgium), at Bruker (Bremen, Germany), and the UHH (Heidelberg, Germany). The conditions under which the strains were grown at the three sites were very similar in incubation parameters (36°C and 5% CO_2_), incubation time (24 h), and growth medium (Columbia agar base medium plus 5% sheep blood [CB + 5% SB]). Remarkably, it was observed that when comparing all the different spectra, they were not clustering per serotype as expected but, rather, formed two distinct clouds on a three-dimensional (3-D) PCA plot, clearly separating Sciensano spectra from UHH spectra (Fig. 5A). This pointed to the existence of one or more variables that had a larger contribution to the total variance of the data set than the individual chemical differences of the different serotypes. After close examination of the exact incubation conditions, it was noticed that the incubation medium originated from two different suppliers (Oxoid versus BD). An experiment was designed to test the alleged influence of the medium supplier. Strains from 8 different serotypes were grown on CB + 5% SB from BD, Oxoid, or bioMérieux and analyzed using FT-IR spectroscopy. When interpreting the 2-D PCA plot, it was observed that the spectra of the strains measured on the Oxoid medium clustered far away from the spectra of the same strains measured on BD and bioMérieux media, regardless of the site at which the spectra were acquired (Fig. 5B). Moreover, measurements of the same reference strain grown at different locations (UHH and Sciensano) and on CB from different suppliers (BD and Oxoid) showed that the influence of the medium supplier is far greater than other interlaboratory differences that might exist (Fig. S1). This indicates that the Oxoid medium contains a component(s) that, presumably indirectly, changes the chemical composition of the sugar-rich outer surface of the pneumococci and that these changes obfuscate the serotype-dependent capsule differences, ultimately complicating the serotyping process when comparing spectra from strains grown on CB + 5% SB plates from different suppliers.

## DISCUSSION

S. pneumoniae is still among the leading causes of infection-induced mortality, especially in developing countries (7). The introduction of PCVs in the early 2000s and their implementation in childhood vaccination programs has greatly decreased the incidence of IPD caused by VT serotypes (19–22). This effect was also initially observed in the unvaccinated adult population in the United States and was attributed to herd immunity (64, 65). However, in recent years, the incidence of IPD worldwide has plateaued or even slightly increased as the result of (i) serotype replacement, since NVT serotypes fill the ecological niche that is left by VT serotypes, and (ii) limitations to herd protection (29, 66–69). As long as there is no vaccine protecting against every pneumococcal strain, independent of serotype, surveillance of circulating serotypes will remain essential in the future and serotyping techniques will continue to be an important tool to monitor pneumococcal seroepidemiology (62, 70).

FT-IR spectroscopy uses infrared light to create absorption spectra of the biological material being probed, yielding information on the vibration and rotation of covalent bonds of the biological structure (41, 42). This technique can detect and discriminate very small chemical differences and was recently tested for serotyping S. pneumoniae (37). Burckhardt et al. (37) used a training set containing the spectra of 120 strains (representing the 24 VT serotypes) to type a set of 168 challenge strains (representing 48 different serotypes, both VT and NVT) and achieved excellent concordance between the results of FT-IR spectroscopy and the quellung reaction. In this study, extensive serotyping validation results for S. pneumoniae were generated using two different approaches (HCA and ANN), surpassing the initial validation study of Burckhardt et al., and promising accuracies were obtained, 98% for predicting training set serotypes and 71.2% for predicting non-training set serotypes.

Interestingly, FT-IR spectroscopy was capable of accurately categorizing all training set strains at the serotype level except for SG24, which could only be classified on the serogroup level. At the time of strain collection, the existence of the new 24C variant (14), which contains a mixture of 24B and 24F repeating units, was not yet known. This finding is relevant because our analysis technique relies on feeding in correct *a priori* information (supervised learning is applied when building both the LDA, grouped by serotype, or the ANN model), and when this condition is not met, the models become highly unreliable. However, it is highly unlikely that the 24B and 24F strains used in our study were “hidden” 24C strains, as 24C strains already show an atypical serological profile when exposed to the routinely used SG24-typing antisera. In this regard, it should be noted that subtyping of SG24 strains using the reference phenotypic methods, such as the quellung reaction and the latex agglutination assay, proves difficult too because of low reactivity with the antisera. Future work will include the incorporation of more SG24 training set strains to increase the robustness of the training set, followed by an in-depth analysis of the measured spectra.

For the prediction of training set serotypes (24 VT and 10 NVT), FT-IR spectroscopy performed very well, with 98% accuracy, and problematic strains seemed to randomly spread out over different serotypes. There could be various reasons explaining the few discordances observed between the results of FT-IR spectroscopy and the quellung reaction. First, although both techniques measure the capsule structure itself, they do this differently. While the quellung reaction uses the 3-D specificity that polyclonal antibodies possess with the pneumococcal capsule, FT-IR spectroscopy probes the vibration and rotation of covalent bonds (typically C-O stretching and O-H bending) both within the pneumococcal outer surface and inside the cell. This implies that some differences between serotypes might be picked up more easily when using the quellung reaction rather than FT-IR spectroscopy and vice versa. Furthermore, FT-IR spectroscopy is not limited to measuring the chemical bonds of the capsule structure, as all C-O and O-H bonds are detected and it can consequently also pick up differences in glycoproteins and/or glycolipids between related strains.

Another reason for the observed discordances could be the limited number of S. pneumoniae strains per serotype (*n *≥ 4) used for training, in combination with large genetic variations for particular serotypes (71–74), possibly translating into small differences between pneumococcal strains belonging to the same serotype. Although a minimum of four strains per serotype was used to build the training set and care was taken to incorporate strains originating from both IPD and NIPD specimens and from two geographical locations (Belgium and The Netherlands), it is possible that the training set was not yet robust enough for certain serotypes. In this regard, it should be noted that there was no WGS information on most of the training set strains and it was thus unknown how much (genetic) variation was represented within the different serotypes in our training set. Currently, work is ongoing to increase the number of strains per serotype in our training set to create a higher degree of robustness.

Finally, we sought to minimize variations in the environmental conditions when growing the pneumococci or measuring them with FT-IR spectroscopy but found them to be unavoidable. It was shown that some degree of variability between measurements of the same strain on different occasions exists and that repeating the measurement with more technical replicates can improve the serotyping accuracy. Furthermore, care was taken to use the growth medium within the mentioned expiration date. However, this still leaves a window of a couple of weeks where the unexpired medium could change slightly and alter the growing conditions, possibly influencing the outcome of the measurement. In this regard, it was found that using CB + 5% SB past the expiration date did alter the serotyping accuracy for certain serotypes (data not shown). Together, the existence of this environmental variation may result in less accurate serotyping and be (partly) responsible for the observed discordances between FT-IR spectroscopy and the quellung reaction.

Given the inherent limitations of the trained models, predicting serotypes not present in the training set (NTS serotypes) and categorizing them as such proved to be challenging, reaching an accuracy of 71.2%. The majority of NTS serotypes that were wrongly typed as training set serotypes had capsule structures that were very similar to some training set serotypes (e.g., 6C versus 6D, 7F versus 7A, 9N versus 9L, and 19A versus 19B), and this appears to be a challenge in our serotyping workflow. It seems unlikely that all these problematic serotypes lack enough discriminatory features in their capsule for the FT-IR spectroscopy to separate them accurately. For example, 19A and 19F are both training set serotypes and can be discriminated accurately using FT-IR spectroscopy, even though their capsule structure is more alike than the 19B capsule structure (8). The nature of the supervised serotyping technique and HCA is to force the categorization of unknown samples within a known data set, and it seems that whenever a serotype is not part of the training set (and our supervised models have not had the opportunity to be trained using that particular serotype), this serotype tends to end up in another serotype cluster based on capsule structure resemblance. This problem can be solved by building extra serogroup-specific ANN models trained exclusively on serotypes with similar capsule structures, thus creating a hierarchical order of ANN models that can be used sequentially to yield the correct serotype. Currently, efforts are being made to incorporate more strains of these problematic serotypes in an extended training set to (i) test whether these serotypes can in fact be discriminated when feeding the supervised models more information and (ii) create more specific ANN models.

Comparison of spectra acquired from strains grown under similar conditions and originating from the same type of IR device (IR Biotyper, Bruker) but measured at different sites was found to be challenging. It was shown that the supplier of CB + 5% SB impacted the comparability of IR spectra between different institutions. More specifically, spectra acquired from strains grown on CB from BD or bioMérieux compared well, as opposed to the spectra measured from strains grown on CB from Oxoid. This observation was independent of the site at which the spectra were measured, suggesting that interlaboratory comparison of spectra was realistic as long as strains were grown on CB from the same supplier. The anticipated variation in composition between the CB media of the different suppliers is currently unknown, and work is ongoing to pinpoint the cause of the observed differences between the different medium suppliers. Future supervised models will be developed to account for this “supplier variation,” enabling the straightforward comparison of IR spectra between laboratories. Interestingly, different broths or the same broth from different suppliers also affect the accuracy of the latex agglutination test (75), suggesting that the challenging comparability between spectra of different laboratories is not solely the result of the FT-IR spectroscopy measuring technique *per se*. At present, FT-IR spectroscopy yields excellent results for accuracy but relies on the construction of a robust in-house training set, a labor-intensive endeavor that is not achievable for every clinical laboratory. However, with the knowledge acquired in this study, the application of a strictly standardized workflow, including defined medium suppliers, should be feasible in the near future.

The results presented herein underline the potential of FT-IR spectroscopy as a serotyping technique for S. pneumoniae. Concordance between FT-IR spectroscopy and the quellung reaction is very high for serotypes that are part of the training set, and future work aims at an all-encompassing, reliable training set. Moreover, FT-IR spectroscopy is a promising technique because of its ease of use, cost effectiveness, and medium-throughput potential, thus making it the ideal candidate to complement the existing phenotypic methods. The workflow of the technique is straightforward and requires a minimal amount of training: Pneumococcal strains grown on blood agar plates are directly applied to a silicon plate using a sterile loop, and the plate is then inserted into the IR Biotyper. The hands-on time is limited to 1 h to 1 h 30 min, and the FT-IR spectroscopy measurement itself takes around 1 h 30 min for a full plate (96 spots). Up to 30 strains can be measured in one measurement run, and the subsequent serotyping analysis workflow described herein should not take longer than 30 min. Consequently, the labor time for serotyping one strain can be as low as 4 min, which is considerably faster than the average time needed to serotype a pneumococcal strain using the quellung reaction (between 5 and 30 min per strain, depending on the specific serotype). Performing an accurate cost comparison between the different phenotypic methods is tricky, mainly because (i) the cost of performing the quellung reaction very much depends on the exact serotype that is being probed and whether a latex agglutination assay is performed too and (ii) the cost of the IR Biotyper machine needs to be added into the equation as a yearly depreciation cost, and this depends on how heavily the machine is used for serotyping pneumococci. Nonetheless, based on the information obtained from the national reference centers involved in this study, the average cost per sample for the quellung reaction was estimated to be around €60. The cost per sample using the IR Biotyper was estimated based on a yearly use of serotyping 1,500 pneumococcal strains (rough average of the yearly number of invasive pneumococci serotyped at the NRC Belgium) and was €20 per sample when using three technical replicates per strain or €60 when using five technical replicates per sample. The majority of strains are accurately serotyped using three technical replicates, and only a subset of strains need to be measured again using five technical replicates, yielding an average cost per sample of around €35. These calculations show that investing in the IR Biotyper machine and using it to serotype pneumococci is cost effective compared to the costs of other phenotypic methods. In this regard, it should be noted that the IR Biotyper has the potential to be used for serotyping other pathogens too, or for outbreak analysis (43–48).

Surprisingly, it was shown that the origin of the growth medium impacts the comparability of IR spectra between different institutions, and more work is required to fully understand the influence of environmental conditions on the IR spectra of pneumococci. In the future, FT-IR spectroscopy has the potential to be used for first-line serotyping (quick, cost effective, and accurate), after which strains that prove more difficult to serotype could be typed using the classical phenotypic or genotypic techniques. In this regard, it should be noted that FT-IR spectroscopy is not dependent on the development of new type-specific antisera and has the potential to quickly detect unknown serotypes. Finally, because certain serotypes are well known to be linked to high antimicrobial resistance, FT-IR spectroscopy could prove useful in clinical laboratory settings in the future, when quick serotyping results could assist clinicians in their decision-making process when considering different treatment options.
