# Supplementary material for: Validation of Fourier Transform Infrared Spectroscopy for Serotyping of Streptococcus pneumoniae
Source: J Clin Microbiol. 2022 Jun 14;60(7):e00325-22. doi: 10.1128/jcm.00325-22 (PMC9297836; doi:10.1128/jcm.00325-22)
Supplement: Supplemental file 1 — Fig. S1 and Tables S1 to S4. Download jcm.00325-22-s0001.pdf, PDF file, 0.9 MB [file jcm.00325-22-s0001.pdf]

Supplemental figures

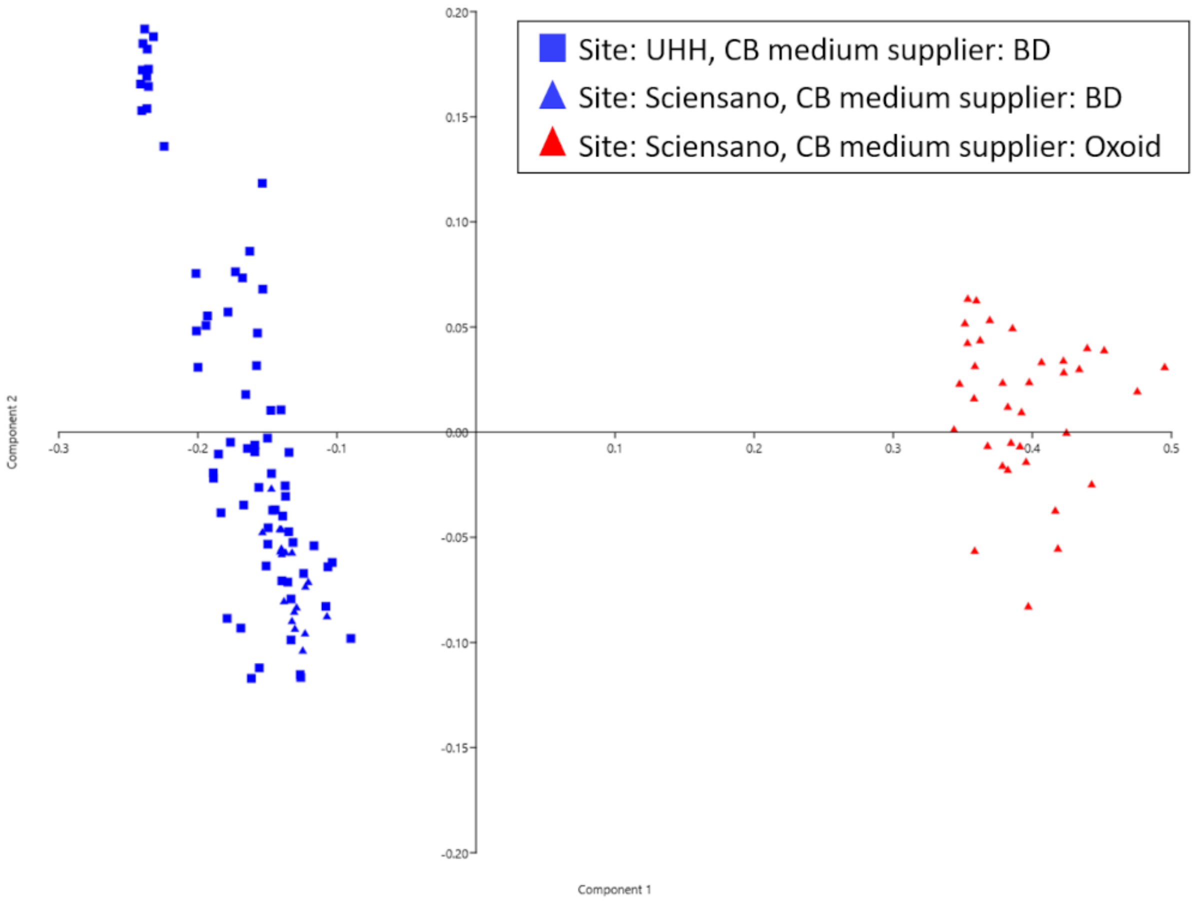

Supplementary Figure 1: 2D-PCA plot showing the results of reference strain ATCC49619 (19F) measured at two sites (UHH and Sciensano) and grown on CB medium from two different suppliers (BD and Oxoid).

Supplemental tables

Supplementary Table 1: List of all pneumococcal strains used in this study.

Isolate	Serotype	Origin	IPD/NIPD isolate	Use of strain
080693	2	NRC for pneumococcal diseases Belgium	IPD isolate	Training set
191024	2	NRC for pneumococcal diseases Belgium	IPD isolate	Training set
200188	3	NRC for pneumococcal diseases Belgium	IPD isolate	Training set
200203	3	NRC for pneumococcal diseases Belgium	IPD isolate	Training set
SP06_F01	3	Sciensano	NIPD isolate	Training set
SP12_A01	3	Sciensano	NIPD isolate	Training set
SP13_A04	3	Sciensano	NIPD isolate	Training set
191445	4	NRC for pneumococcal diseases Belgium	IPD isolate	Training set
191447	4	NRC for pneumococcal diseases Belgium	IPD isolate	Training set
200614	4	NRC for pneumococcal diseases Belgium	IPD isolate	Training set
171076	5	NRC for pneumococcal diseases Belgium	IPD isolate	Training set
180124	5	NRC for pneumococcal diseases Belgium	IPD isolate	Training set
200189	8	NRC for pneumococcal diseases Belgium	IPD isolate	Training set
200191	8	NRC for pneumococcal diseases Belgium	IPD isolate	Training set
200209	8	NRC for pneumococcal diseases Belgium	IPD isolate	Training set
200220	14	NRC for pneumococcal diseases Belgium	IPD isolate	Training set
200225	14	NRC for pneumococcal diseases Belgium	IPD isolate	Training set
SP12_A11	14	Sciensano	NIPD isolate	Training set
SP13_B07	14	Sciensano	NIPD isolate	Training set
SP13_C04	14	Sciensano	NIPD isolate	Training set
SP13_C15	14	Sciensano	NIPD isolate	Training set
SP13_F13	14	Sciensano	NIPD isolate	Training set
200385	20	NRC for pneumococcal diseases Belgium	IPD isolate	Training set

200496	20	NRC for pneumococcal diseases Belgium	IPD isolate	Training set
200680	20	NRC for pneumococcal diseases Belgium	IPD isolate	Training set
191569	38	NRC for pneumococcal diseases Belgium	IPD isolate	Training set
191649	38	NRC for pneumococcal diseases Belgium	IPD isolate	Training set
191506	10A	NRC for pneumococcal diseases Belgium	IPD isolate	Training set
191543	10A	NRC for pneumococcal diseases Belgium	IPD isolate	Training set
200657	10A	NRC for pneumococcal diseases Belgium	IPD isolate	Training set
SP14_E14	11A	Sciensano	NIPD isolate	Training set
SP14_G04	11A	Sciensano	NIPD isolate	Training set
SP14_K09	11A	Sciensano	NIPD isolate	Training set
SP14_L25	11A	Sciensano	NIPD isolate	Training set
200205	12F	NRC for pneumococcal diseases Belgium	IPD isolate	Training set
200212	12F	NRC for pneumococcal diseases Belgium	IPD isolate	Training set
200704	12F	NRC for pneumococcal diseases Belgium	IPD isolate	Training set
SP13_C13	12F	Sciensano	NIPD isolate	Training set
200219	15A	NRC for pneumococcal diseases Belgium	IPD isolate	Training set
200222	15A	NRC for pneumococcal diseases Belgium	IPD isolate	Training set
SP12_A05	15A	Sciensano	NIPD isolate	Training set
SP13_A12	15A	Sciensano	NIPD isolate	Training set
SP13_C05	15A	Sciensano	NIPD isolate	Training set
SP14_A39	15A	Sciensano	NIPD isolate	Training set
180465	15B	NRC for pneumococcal diseases Belgium	IPD isolate	Training set
180578	15B	NRC for pneumococcal diseases Belgium	IPD isolate	Training set
191311	15B	NRC for pneumococcal diseases Belgium	IPD isolate	Training set
SP12_I13	15B	Sciensano	NIPD isolate	Training set
SP13_K18	15B	Sciensano	NIPD isolate	Training set
SP13_L29	15B	Sciensano	NIPD isolate	Training set
SP14_K17	15B	Sciensano	NIPD isolate	Training set
200201	16F	NRC for pneumococcal diseases Belgium	IPD isolate	Training set

200242	16F	NRC for pneumococcal diseases Belgium	IPD isolate	Training set
200662	16F	NRC for pneumococcal diseases Belgium	IPD isolate	Training set
190218	17F	NRC for pneumococcal diseases Belgium	IPD isolate	Training set
200398	17F	NRC for pneumococcal diseases Belgium	IPD isolate	Training set
SP13_J15	17F	Sciensano	NIPD isolate	Training set
190411	18C	NRC for pneumococcal diseases Belgium	IPD isolate	Training set
190957	18C	NRC for pneumococcal diseases Belgium	IPD isolate	Training set
191292	18C	NRC for pneumococcal diseases Belgium	IPD isolate	Training set
SP14_C01	19A	Sciensano	NIPD isolate	Training set
SP14_E02	19A	Sciensano	NIPD isolate	Training set
SP14_H04	19A	Sciensano	NIPD isolate	Training set
SP14_K01	19A	Sciensano	NIPD isolate	Training set
200396	19F	NRC for pneumococcal diseases Belgium	IPD isolate	Training set
ATCC49619 R2/51	19F	NRC for pneumococcal diseases Belgium	IPD isolate	Training set
SP13_A22	19F	Sciensano	NIPD isolate	Training set
SP13_B23	19F	Sciensano	NIPD isolate	Training set
SP14_E19	19F	Sciensano	NIPD isolate	Training set
SP14_H01	19F	Sciensano	NIPD isolate	Training set
SP14_K24	19F	Sciensano	NIPD isolate	Training set
200202	22F	NRC for pneumococcal diseases Belgium	IPD isolate	Training set
200233	22F	NRC for pneumococcal diseases Belgium	IPD isolate	Training set
200648	22F	NRC for pneumococcal diseases Belgium	IPD isolate	Training set
191624	23A	NRC for pneumococcal diseases Belgium	IPD isolate	Training set
191642	23A	NRC for pneumococcal diseases Belgium	IPD isolate	Training set
200473	23A	NRC for pneumococcal diseases Belgium	IPD isolate	Training set
200605	23A	NRC for pneumococcal diseases Belgium	IPD isolate	Training set
SP13_D20	23B	Sciensano	NIPD isolate	Training set
SP14_D14	23B	Sciensano	NIPD isolate	Training set
SP14_G06	23B	Sciensano	NIPD isolate	Training set

SP14_K18	23B	Sciensano	NIPD isolate	Training set
SP14_M03	23B	Sciensano	NIPD isolate	Training set
110270	23F	NRC for pneumococcal diseases Belgium	IPD isolate	Training set
180504	23F	NRC for pneumococcal diseases Belgium	IPD isolate	Training set
180822	23F	NRC for pneumococcal diseases Belgium	IPD isolate	Training set
SP13_L32	23F	Sciensano	NIPD isolate	Training set
160649	24A	NRC for pneumococcal diseases Belgium	IPD isolate	Training set
191429	24A	NRC for pneumococcal diseases Belgium	IPD isolate	Training set
190739	24B	NRC for pneumococcal diseases Belgium	IPD isolate	Training set
191589	24B	NRC for pneumococcal diseases Belgium	IPD isolate	Training set
191347	24F	NRC for pneumococcal diseases Belgium	IPD isolate	Training set
191398	24F	NRC for pneumococcal diseases Belgium	IPD isolate	Training set
SP08_H09	24F	Sciensano	NIPD isolate	Training set
SP13_C26	24F	Sciensano	NIPD isolate	Training set
SP14_E24	24F	Sciensano	NIPD isolate	Training set
200236	33F	NRC for pneumococcal diseases Belgium	IPD isolate	Training set
200241	33F	NRC for pneumococcal diseases Belgium	IPD isolate	Training set
200529	33F	NRC for pneumococcal diseases Belgium	IPD isolate	Training set
200619	33F	NRC for pneumococcal diseases Belgium	IPD isolate	Training set
191274	35B	NRC for pneumococcal diseases Belgium	IPD isolate	Training set
200409	35B	NRC for pneumococcal diseases Belgium	IPD isolate	Training set
SP14_A04	35B	Sciensano	NIPD isolate	Training set
SP14_A14	35B	Sciensano	NIPD isolate	Training set
SP14_C08	35B	Sciensano	NIPD isolate	Training set
SP14_L21	35B	Sciensano	NIPD isolate	Training set
SP14_M02	35B	Sciensano	NIPD isolate	Training set
191417	6A	NRC for pneumococcal diseases Belgium	IPD isolate	Training set
200389	6A	NRC for pneumococcal diseases Belgium	IPD isolate	Training set
SP12_I38	6A	Sciensano	NIPD isolate	Training set

SP13_M04	6A	Sciensano	NIPD isolate	Training set
160886	6B	NRC for pneumococcal diseases Belgium	IPD isolate	Training set
180659	6B	NRC for pneumococcal diseases Belgium	IPD isolate	Training set
190736	6B	NRC for pneumococcal diseases Belgium	IPD isolate	Training set
SP12_I28	6B	Sciensano	NIPD isolate	Training set
200200	6C	NRC for pneumococcal diseases Belgium	IPD isolate	Training set
5DCC053-03	6C	NRC for pneumococcal diseases Belgium	IPD isolate	Training set
SP12_G05	6C	Sciensano	NIPD isolate	Training set
SP13_A39	6C	Sciensano	NIPD isolate	Training set
SP13_B21	6C	Sciensano	NIPD isolate	Training set
SP13_E17	6C	Sciensano	NIPD isolate	Training set
SP14_J11	6C	Sciensano	NIPD isolate	Training set
SP14_J35	6C	Sciensano	NIPD isolate	Training set
191102	7F	NRC for pneumococcal diseases Belgium	IPD isolate	Training set
191278	7F	NRC for pneumococcal diseases Belgium	IPD isolate	Training set
191588	7F	NRC for pneumococcal diseases Belgium	IPD isolate	Training set
200381	7F	NRC for pneumococcal diseases Belgium	IPD isolate	Training set
191622	9N	NRC for pneumococcal diseases Belgium	IPD isolate	Training set
200404	9N	NRC for pneumococcal diseases Belgium	IPD isolate	Training set
200700	9N	NRC for pneumococcal diseases Belgium	IPD isolate	Training set
190022	9V	NRC for pneumococcal diseases Belgium	IPD isolate	Training set
190789	9V	NRC for pneumococcal diseases Belgium	IPD isolate	Training set
191086	9V	NRC for pneumococcal diseases Belgium	IPD isolate	Training set
SP10_I04	9V	Sciensano	NIPD isolate	Training set
2180060	1	NRC for pneumococcal diseases The Netherlands	IPD isolate	Training set + Optimizing PneumoClassifier
2193153	1	NRC for pneumococcal diseases The Netherlands	IPD isolate	Training set + Optimizing PneumoClassifier
2193331	1	NRC for pneumococcal diseases The Netherlands	IPD isolate	Training set + Optimizing PneumoClassifier
2021863	2	NRC for pneumococcal diseases The Netherlands	IPD isolate	Training set + Optimizing PneumoClassifier
2170441	2	NRC for pneumococcal diseases The Netherlands	IPD isolate	Training set + Optimizing PneumoClassifier

2180118	3	NRC for pneumococcal diseases The Netherlands	IPD isolate	Training set + Optimizing PneumoClassifier
2193015	3	NRC for pneumococcal diseases The Netherlands	IPD isolate	Training set + Optimizing PneumoClassifier
2201551	3	NRC for pneumococcal diseases The Netherlands	IPD isolate	Training set + Optimizing PneumoClassifier
2193190	4	NRC for pneumococcal diseases The Netherlands	IPD isolate	Training set + Optimizing PneumoClassifier
2120335	5	NRC for pneumococcal diseases The Netherlands	IPD isolate	Training set + Optimizing PneumoClassifier
2180589	8	NRC for pneumococcal diseases The Netherlands	IPD isolate	Training set + Optimizing PneumoClassifier
2201794	8	NRC for pneumococcal diseases The Netherlands	IPD isolate	Training set + Optimizing PneumoClassifier
2180086	14	NRC for pneumococcal diseases The Netherlands	IPD isolate	Training set + Optimizing PneumoClassifier
2193249	14	NRC for pneumococcal diseases The Netherlands	IPD isolate	Training set + Optimizing PneumoClassifier
2180340	20	NRC for pneumococcal diseases The Netherlands	IPD isolate	Training set + Optimizing PneumoClassifier
2180322	38	NRC for pneumococcal diseases The Netherlands	IPD isolate	Training set + Optimizing PneumoClassifier
2193223	38	NRC for pneumococcal diseases The Netherlands	IPD isolate	Training set + Optimizing PneumoClassifier
2180110	10A	NRC for pneumococcal diseases The Netherlands	IPD isolate	Training set + Optimizing PneumoClassifier
2180009	11A	NRC for pneumococcal diseases The Netherlands	IPD isolate	Training set + Optimizing PneumoClassifier
2193403	11A	NRC for pneumococcal diseases The Netherlands	IPD isolate	Training set + Optimizing PneumoClassifier
2180135	12F	NRC for pneumococcal diseases The Netherlands	IPD isolate	Training set + Optimizing PneumoClassifier
2180106	15A	NRC for pneumococcal diseases The Netherlands	IPD isolate	Training set + Optimizing PneumoClassifier
2193058	15A	NRC for pneumococcal diseases The Netherlands	IPD isolate	Training set + Optimizing PneumoClassifier
2180052	15B	NRC for pneumococcal diseases The Netherlands	IPD isolate	Training set + Optimizing PneumoClassifier
2193286	15B	NRC for pneumococcal diseases The Netherlands	IPD isolate	Training set + Optimizing PneumoClassifier
2180207	16F	NRC for pneumococcal diseases The Netherlands	IPD isolate	Training set + Optimizing PneumoClassifier
2193231	16F	NRC for pneumococcal diseases The Netherlands	IPD isolate	Training set + Optimizing PneumoClassifier
2193238	17F	NRC for pneumococcal diseases The Netherlands	IPD isolate	Training set + Optimizing PneumoClassifier
2201606	17F	NRC for pneumococcal diseases The Netherlands	IPD isolate	Training set + Optimizing PneumoClassifier
2180436	18C	NRC for pneumococcal diseases The Netherlands	IPD isolate	Training set + Optimizing PneumoClassifier
2193440	19A	NRC for pneumococcal diseases The Netherlands	IPD isolate	Training set + Optimizing PneumoClassifier
2201734	19A	NRC for pneumococcal diseases The Netherlands	IPD isolate	Training set + Optimizing PneumoClassifier
2180426	19F	NRC for pneumococcal diseases The Netherlands	IPD isolate	Training set + Optimizing PneumoClassifier
2193221	19F	NRC for pneumococcal diseases The Netherlands	IPD isolate	Training set + Optimizing PneumoClassifier

2201773	19F	NRC for pneumococcal diseases The Netherlands	IPD isolate	Training set + Optimizing PneumoClassifier
2201627	22F	NRC for pneumococcal diseases The Netherlands	IPD isolate	Training set + Optimizing PneumoClassifier
2201604	23A	NRC for pneumococcal diseases The Netherlands	IPD isolate	Training set + Optimizing PneumoClassifier
2193198	23B	NRC for pneumococcal diseases The Netherlands	IPD isolate	Training set + Optimizing PneumoClassifier
2201448	23B	NRC for pneumococcal diseases The Netherlands	IPD isolate	Training set + Optimizing PneumoClassifier
2180071	23F	NRC for pneumococcal diseases The Netherlands	IPD isolate	Training set + Optimizing PneumoClassifier
2170444	24A	NRC for pneumococcal diseases The Netherlands	IPD isolate	Training set + Optimizing PneumoClassifier
2120411	24B	NRC for pneumococcal diseases The Netherlands	IPD isolate	Training set + Optimizing PneumoClassifier
2121676	24B	NRC for pneumococcal diseases The Netherlands	IPD isolate	Training set + Optimizing PneumoClassifier
2180348	24F	NRC for pneumococcal diseases The Netherlands	IPD isolate	Training set + Optimizing PneumoClassifier
2193083	24F	NRC for pneumococcal diseases The Netherlands	IPD isolate	Training set + Optimizing PneumoClassifier
2201638	33F	NRC for pneumococcal diseases The Netherlands	IPD isolate	Training set + Optimizing PneumoClassifier
2180276	35B	NRC for pneumococcal diseases The Netherlands	IPD isolate	Training set + Optimizing PneumoClassifier
2201740	35B	NRC for pneumococcal diseases The Netherlands	IPD isolate	Training set + Optimizing PneumoClassifier
2180226	6A	NRC for pneumococcal diseases The Netherlands	IPD isolate	Training set + Optimizing PneumoClassifier
2193076	6A	NRC for pneumococcal diseases The Netherlands	IPD isolate	Training set + Optimizing PneumoClassifier
2201720	6A	NRC for pneumococcal diseases The Netherlands	IPD isolate	Training set + Optimizing PneumoClassifier
2193159	6B	NRC for pneumococcal diseases The Netherlands	IPD isolate	Training set + Optimizing PneumoClassifier
2180284	6C	NRC for pneumococcal diseases The Netherlands	IPD isolate	Training set + Optimizing PneumoClassifier
2201527	6C	NRC for pneumococcal diseases The Netherlands	IPD isolate	Training set + Optimizing PneumoClassifier
2180250	7F	NRC for pneumococcal diseases The Netherlands	IPD isolate	Training set + Optimizing PneumoClassifier
2193275	7F	NRC for pneumococcal diseases The Netherlands	IPD isolate	Training set + Optimizing PneumoClassifier
2180286	9N	NRC for pneumococcal diseases The Netherlands	IPD isolate	Training set + Optimizing PneumoClassifier
2193165	9N	NRC for pneumococcal diseases The Netherlands	IPD isolate	Training set + Optimizing PneumoClassifier
2180603	9V	NRC for pneumococcal diseases The Netherlands	IPD isolate	Training set + Optimizing PneumoClassifier
2180637	9V	NRC for pneumococcal diseases The Netherlands	IPD isolate	Training set + Optimizing PneumoClassifier
ATCC33400 R5/31,32,33,34	1	NRC for pneumococcal diseases Belgium	IPD isolate	Training set + Testing robustness
191596	2	NRC for pneumococcal diseases Belgium	IPD isolate	Training set + Testing robustness

200228	3	NRC for pneumococcal diseases Belgium	IPD isolate	Training set + Testing robustness
200195	4	NRC for pneumococcal diseases Belgium	IPD isolate	Training set + Testing robustness
181203	5	NRC for pneumococcal diseases Belgium	IPD isolate	Training set + Testing robustness
200155	14	NRC for pneumococcal diseases Belgium	IPD isolate	Training set + Testing robustness
191369	20	NRC for pneumococcal diseases Belgium	IPD isolate	Training set + Testing robustness
191073	38	NRC for pneumococcal diseases Belgium	IPD isolate	Training set + Testing robustness
200374	10A	NRC for pneumococcal diseases Belgium	IPD isolate	Training set + Testing robustness
200185	11A	NRC for pneumococcal diseases Belgium	IPD isolate	Training set + Testing robustness
200204	11A	NRC for pneumococcal diseases Belgium	IPD isolate	Training set + Testing robustness
5DCC098-08	11A	NRC for pneumococcal diseases Belgium	IPD isolate	Training set + Testing robustness
200196	15A	NRC for pneumococcal diseases Belgium	IPD isolate	Training set + Testing robustness
191134	15B	NRC for pneumococcal diseases Belgium	IPD isolate	Training set + Testing robustness
200160	16F	NRC for pneumococcal diseases Belgium	IPD isolate	Training set + Testing robustness
200215	17F	NRC for pneumococcal diseases Belgium	IPD isolate	Training set + Testing robustness
200262	18C	NRC for pneumococcal diseases Belgium	IPD isolate	Training set + Testing robustness
200156	19A	NRC for pneumococcal diseases Belgium	IPD isolate	Training set + Testing robustness
200186	19A	NRC for pneumococcal diseases Belgium	IPD isolate	Training set + Testing robustness
200193	19A	NRC for pneumococcal diseases Belgium	IPD isolate	Training set + Testing robustness
191453	19F	NRC for pneumococcal diseases Belgium	IPD isolate	Training set + Testing robustness
200192	22F	NRC for pneumococcal diseases Belgium	IPD isolate	Training set + Testing robustness
200199	22F	NRC for pneumococcal diseases Belgium	IPD isolate	Training set + Testing robustness
191308	23A	NRC for pneumococcal diseases Belgium	IPD isolate	Training set + Testing robustness
200274	23A	NRC for pneumococcal diseases Belgium	IPD isolate	Training set + Testing robustness
200187	23B	NRC for pneumococcal diseases Belgium	IPD isolate	Training set + Testing robustness
200210	23B	NRC for pneumococcal diseases Belgium	IPD isolate	Training set + Testing robustness
5DCC036-02	23B	NRC for pneumococcal diseases Belgium	IPD isolate	Training set + Testing robustness
200397	24A	NRC for pneumococcal diseases Belgium	IPD isolate	Training set + Testing robustness
160623	24B	NRC for pneumococcal diseases Belgium	IPD isolate	Training set + Testing robustness
191270	24F	NRC for pneumococcal diseases Belgium	IPD isolate	Training set + Testing robustness

200207	33F	NRC for pneumococcal diseases Belgium	IPD isolate	Training set + Testing robustness
200194	35B	NRC for pneumococcal diseases Belgium	IPD isolate	Training set + Testing robustness
191399	6A	NRC for pneumococcal diseases Belgium	IPD isolate	Training set + Testing robustness
190154	6B	NRC for pneumococcal diseases Belgium	IPD isolate	Training set + Testing robustness
200198	6C	NRC for pneumococcal diseases Belgium	IPD isolate	Training set + Testing robustness
191406	7F	NRC for pneumococcal diseases Belgium	IPD isolate	Training set + Testing robustness
200379	9N	NRC for pneumococcal diseases Belgium	IPD isolate	Training set + Testing robustness
200322	9V	NRC for pneumococcal diseases Belgium	IPD isolate	Training set + Testing robustness
190523	1	NRC for pneumococcal diseases Belgium	IPD isolate	Validation set
871332	1	NRC for pneumococcal diseases Belgium	IPD isolate	Validation set
871349	1	NRC for pneumococcal diseases Belgium	IPD isolate	Validation set
SSI 89	1	NRC for pneumococcal diseases Denmark	IPD isolate	Validation set
SSI 105	2	NRC for pneumococcal diseases Denmark	IPD isolate	Validation set
SSI 106	2	NRC for pneumococcal diseases Denmark	IPD isolate	Validation set
SSI 107	2	NRC for pneumococcal diseases Denmark	IPD isolate	Validation set
SSI 108	2	NRC for pneumococcal diseases Denmark	IPD isolate	Validation set
SSI 109	2	NRC for pneumococcal diseases Denmark	IPD isolate	Validation set
SSI 1	3	NRC for pneumococcal diseases Denmark	IPD isolate	Validation set
SSI 26	3	NRC for pneumococcal diseases Denmark	IPD isolate	Validation set
SSI 46	3	NRC for pneumococcal diseases Denmark	IPD isolate	Validation set
SSI 66	3	NRC for pneumococcal diseases Denmark	IPD isolate	Validation set
2200309	4	NRC for pneumococcal diseases The Netherlands	IPD isolate	Validation set
2200397	4	NRC for pneumococcal diseases The Netherlands	IPD isolate	Validation set
2200399	4	NRC for pneumococcal diseases The Netherlands	IPD isolate	Validation set
PN-20-251	4	NRC for pneumococcal diseases Belgium	IPD isolate	Validation set
SSI 90	4	NRC for pneumococcal diseases Denmark	IPD isolate	Validation set
SSI 99	4	NRC for pneumococcal diseases Denmark	IPD isolate	Validation set
170525	5	NRC for pneumococcal diseases Belgium	IPD isolate	Validation set
2141388	5	NRC for pneumococcal diseases The Netherlands	IPD isolate	Validation set

2161051	5	NRC for pneumococcal diseases The Netherlands	IPD isolate	Validation set
2171674	5	NRC for pneumococcal diseases The Netherlands	IPD isolate	Validation set
SSI 27	8	NRC for pneumococcal diseases Denmark	IPD isolate	Validation set
SSI 3	8	NRC for pneumococcal diseases Denmark	IPD isolate	Validation set
SSI 47	8	NRC for pneumococcal diseases Denmark	IPD isolate	Validation set
SSI 67	8	NRC for pneumococcal diseases Denmark	IPD isolate	Validation set
2050174	13	NRC for pneumococcal diseases The Netherlands	IPD isolate	Validation set
SP05_L20	13	Sciensano	NIPD isolate	Validation set
SP08_D13	13	Sciensano	NIPD isolate	Validation set
200530	14	NRC for pneumococcal diseases Belgium	IPD isolate	Validation set
2201068	14	NRC for pneumococcal diseases The Netherlands	IPD isolate	Validation set
2201112	14	NRC for pneumococcal diseases The Netherlands	IPD isolate	Validation set
2201562	14	NRC for pneumococcal diseases The Netherlands	IPD isolate	Validation set
2201563	14	NRC for pneumococcal diseases The Netherlands	IPD isolate	Validation set
SSI 9	14	NRC for pneumococcal diseases Denmark	IPD isolate	Validation set
SSI 12	20	NRC for pneumococcal diseases Denmark	IPD isolate	Validation set
SSI 33	20	NRC for pneumococcal diseases Denmark	IPD isolate	Validation set
SSI 53	20	NRC for pneumococcal diseases Denmark	IPD isolate	Validation set
SSI 73	20	NRC for pneumococcal diseases Denmark	IPD isolate	Validation set
2182834	21	NRC for pneumococcal diseases The Netherlands	IPD isolate	Validation set
SP05_I02	21	Sciensano	NIPD isolate	Validation set
SP10_K12	21	Sciensano	NIPD isolate	Validation set
SP10_O27	21	Sciensano	NIPD isolate	Validation set
2191927	27	NRC for pneumococcal diseases The Netherlands	IPD isolate	Validation set
2041198	29	NRC for pneumococcal diseases The Netherlands	IPD isolate	Validation set
2182594	29	NRC for pneumococcal diseases The Netherlands	IPD isolate	Validation set
2192942	29	NRC for pneumococcal diseases The Netherlands	IPD isolate	Validation set
2192942	29	NRC for pneumococcal diseases The Netherlands	IPD isolate	Validation set
2190712	31	NRC for pneumococcal diseases The Netherlands	IPD isolate	Validation set

SP05_H17	31	Sciensano	NIPD isolate	Validation set
SP07_A30	31	Sciensano	NIPD isolate	Validation set
SP07_E07	31	Sciensano	NIPD isolate	Validation set
SP08_F03	31	Sciensano	NIPD isolate	Validation set
2192809	34	NRC for pneumococcal diseases The Netherlands	IPD isolate	Validation set
SP05_B36	34	Sciensano	NIPD isolate	Validation set
SP05_L15	34	Sciensano	NIPD isolate	Validation set
SP05_O22	34	Sciensano	NIPD isolate	Validation set
SP07_M18	34	Sciensano	NIPD isolate	Validation set
SP10_D11	34	Sciensano	NIPD isolate	Validation set
921311	36	NRC for pneumococcal diseases The Netherlands	IPD isolate	Validation set
950665	37	NRC for pneumococcal diseases The Netherlands	IPD isolate	Validation set
2200147	38	NRC for pneumococcal diseases The Netherlands	IPD isolate	Validation set
SSI 25	38	NRC for pneumococcal diseases Denmark	IPD isolate	Validation set
SSI 45	38	NRC for pneumococcal diseases Denmark	IPD isolate	Validation set
SSI 65	38	NRC for pneumococcal diseases Denmark	IPD isolate	Validation set
171180	39	NRC for pneumococcal diseases Belgium	IPD isolate	Validation set
901178	39	NRC for pneumococcal diseases The Netherlands	IPD isolate	Validation set
960713	39	NRC for pneumococcal diseases The Netherlands	IPD isolate	Validation set
2011892	40	NRC for pneumococcal diseases The Netherlands	IPD isolate	Validation set
2020865	40	NRC for pneumococcal diseases The Netherlands	IPD isolate	Validation set
2021042	40	NRC for pneumococcal diseases The Netherlands	IPD isolate	Validation set
2051098	40	NRC for pneumococcal diseases The Netherlands	IPD isolate	Validation set
2131765	40	NRC for pneumococcal diseases The Netherlands	IPD isolate	Validation set
2021231	42	NRC for pneumococcal diseases The Netherlands	IPD isolate	Validation set
851120	43	NRC for pneumococcal diseases The Netherlands	IPD isolate	Validation set
2071855	44	NRC for pneumococcal diseases The Netherlands	IPD isolate	Validation set
2071856	45	NRC for pneumococcal diseases The Netherlands	IPD isolate	Validation set
930225	46	NRC for pneumococcal diseases The Netherlands	IPD isolate	Validation set

2010281	48	NRC for pneumococcal diseases The Netherlands	IPD isolate	Validation set
SSI 29	10A	NRC for pneumococcal diseases Denmark	IPD isolate	Validation set
SSI 49	10A	NRC for pneumococcal diseases Denmark	IPD isolate	Validation set
SSI 6	10A	NRC for pneumococcal diseases Denmark	IPD isolate	Validation set
SSI 69	10A	NRC for pneumococcal diseases Denmark	IPD isolate	Validation set
2192118	10B	NRC for pneumococcal diseases The Netherlands	IPD isolate	Validation set
2070888	10C	NRC for pneumococcal diseases The Netherlands	IPD isolate	Validation set
2031652	10F	NRC for pneumococcal diseases The Netherlands	IPD isolate	Validation set
SSI 30	11A	NRC for pneumococcal diseases Denmark	IPD isolate	Validation set
SSI 50	11A	NRC for pneumococcal diseases Denmark	IPD isolate	Validation set
SSI 7	11A	NRC for pneumococcal diseases Denmark	IPD isolate	Validation set
SSI 70	11A	NRC for pneumococcal diseases Denmark	IPD isolate	Validation set
2070400	11B	NRC for pneumococcal diseases The Netherlands	IPD isolate	Validation set
2070889	11C	NRC for pneumococcal diseases The Netherlands	IPD isolate	Validation set
2190913	11D	NRC for pneumococcal diseases The Netherlands	IPD isolate	Validation set
2191286	11D	NRC for pneumococcal diseases The Netherlands	IPD isolate	Validation set
2191621	11D	NRC for pneumococcal diseases The Netherlands	IPD isolate	Validation set
2193044	11D	NRC for pneumococcal diseases The Netherlands	IPD isolate	Validation set
2193103	11D	NRC for pneumococcal diseases The Netherlands	IPD isolate	Validation set
2070891	11F	NRC for pneumococcal diseases The Netherlands	IPD isolate	Validation set
2031266	12A	NRC for pneumococcal diseases The Netherlands	IPD isolate	Validation set
200428	12B	NRC for pneumococcal diseases Belgium	IPD isolate	Validation set
2070892	12B	NRC for pneumococcal diseases The Netherlands	IPD isolate	Validation set
SSI 31	12F	NRC for pneumococcal diseases Denmark	IPD isolate	Validation set
SSI 51	12F	NRC for pneumococcal diseases Denmark	IPD isolate	Validation set
SSI 71	12F	NRC for pneumococcal diseases Denmark	IPD isolate	Validation set
SSI 8	12F	NRC for pneumococcal diseases Denmark	IPD isolate	Validation set
SSI 19	15A	NRC for pneumococcal diseases Denmark	IPD isolate	Validation set
SSI 39	15A	NRC for pneumococcal diseases Denmark	IPD isolate	Validation set

SSI 59	15A	NRC for pneumococcal diseases Denmark	IPD isolate	Validation set
SSI 78	15A	NRC for pneumococcal diseases Denmark	IPD isolate	Validation set
SSI 13	15B	NRC for pneumococcal diseases Denmark	IPD isolate	Validation set
SSI 34	15B	NRC for pneumococcal diseases Denmark	IPD isolate	Validation set
SSI 54	15B	NRC for pneumococcal diseases Denmark	IPD isolate	Validation set
SSI 74	15B	NRC for pneumococcal diseases Denmark	IPD isolate	Validation set
200534	15C	NRC for pneumococcal diseases Belgium	IPD isolate	Validation set
2193439	15C	NRC for pneumococcal diseases The Netherlands	IPD isolate	Validation set
SSI 101	15C	NRC for pneumococcal diseases Denmark	IPD isolate	Validation set
SSI 103	15C	NRC for pneumococcal diseases Denmark	IPD isolate	Validation set
SSI 104	15C	NRC for pneumococcal diseases Denmark	IPD isolate	Validation set
SSI 84	15C	NRC for pneumococcal diseases Denmark	IPD isolate	Validation set
SSI 97	15C	NRC for pneumococcal diseases Denmark	IPD isolate	Validation set
200589	15F	NRC for pneumococcal diseases Belgium	IPD isolate	Validation set
2130344	15F	NRC for pneumococcal diseases The Netherlands	IPD isolate	Validation set
2160644	15F	NRC for pneumococcal diseases The Netherlands	IPD isolate	Validation set
2070893	16A	NRC for pneumococcal diseases The Netherlands	IPD isolate	Validation set
SSI 23	16F	NRC for pneumococcal diseases Denmark	IPD isolate	Validation set
SSI 43	16F	NRC for pneumococcal diseases Denmark	IPD isolate	Validation set
SSI 63	16F	NRC for pneumococcal diseases Denmark	IPD isolate	Validation set
SSI 82	16F	NRC for pneumococcal diseases Denmark	IPD isolate	Validation set
SSI 102	17A	NRC for pneumococcal diseases Denmark	IPD isolate	Validation set
SSI 87	17A	NRC for pneumococcal diseases Denmark	IPD isolate	Validation set
SSI 98	17A	NRC for pneumococcal diseases Denmark	IPD isolate	Validation set
SSI 10	17F	NRC for pneumococcal diseases Denmark	IPD isolate	Validation set
SSI 52	17F	NRC for pneumococcal diseases Denmark	IPD isolate	Validation set
SSI 72	17F	NRC for pneumococcal diseases Denmark	IPD isolate	Validation set
SSI 96	17F	NRC for pneumococcal diseases Denmark	IPD isolate	Validation set
191555	18A	NRC for pneumococcal diseases Belgium	IPD isolate	Validation set

2094119	18A	NRC for pneumococcal diseases The Netherlands	IPD isolate	Validation set
2100138	18A	NRC for pneumococcal diseases The Netherlands	IPD isolate	Validation set
2111480	18A	NRC for pneumococcal diseases The Netherlands	IPD isolate	Validation set
2190011	18A	NRC for pneumococcal diseases The Netherlands	IPD isolate	Validation set
2190869	18A	NRC for pneumococcal diseases The Netherlands	IPD isolate	Validation set
2120211	18B	NRC for pneumococcal diseases The Netherlands	IPD isolate	Validation set
2191603	18C	NRC for pneumococcal diseases The Netherlands	IPD isolate	Validation set
2192795	18C	NRC for pneumococcal diseases The Netherlands	IPD isolate	Validation set
2202092	18C	NRC for pneumococcal diseases The Netherlands	IPD isolate	Validation set
2202190	18C	NRC for pneumococcal diseases The Netherlands	IPD isolate	Validation set
PN-20-235	18C	NRC for pneumococcal diseases Belgium	IPD isolate	Validation set
SSI 11	18C	NRC for pneumococcal diseases Denmark	IPD isolate	Validation set
2150080	18F	NRC for pneumococcal diseases The Netherlands	IPD isolate	Validation set
SSI 14	19A	NRC for pneumococcal diseases Denmark	IPD isolate	Validation set
SSI 15	19A	NRC for pneumococcal diseases Denmark	IPD isolate	Validation set
SSI 35	19A	NRC for pneumococcal diseases Denmark	IPD isolate	Validation set
SSI 55	19A	NRC for pneumococcal diseases Denmark	IPD isolate	Validation set
SSI 75	19A	NRC for pneumococcal diseases Denmark	IPD isolate	Validation set
170723	19B	NRC for pneumococcal diseases Belgium	IPD isolate	Validation set
840749	19B	NRC for pneumococcal diseases The Netherlands	IPD isolate	Validation set
871300	19B	NRC for pneumococcal diseases The Netherlands	IPD isolate	Validation set
880965	19B	NRC for pneumococcal diseases The Netherlands	IPD isolate	Validation set
2070894	19C	NRC for pneumococcal diseases The Netherlands	IPD isolate	Validation set
2202207	19F	NRC for pneumococcal diseases The Netherlands	IPD isolate	Validation set
2202274	19F	NRC for pneumococcal diseases The Netherlands	IPD isolate	Validation set
2202422	19F	NRC for pneumococcal diseases The Netherlands	IPD isolate	Validation set
2202440	19F	NRC for pneumococcal diseases The Netherlands	IPD isolate	Validation set
PN-20-34	19F	NRC for pneumococcal diseases Belgium	IPD isolate	Validation set
SSI 100	19F	NRC for pneumococcal diseases Denmark	IPD isolate	Validation set

SSI 92	19F	NRC for pneumococcal diseases Denmark	IPD isolate	Validation set
2150795	22A	NRC for pneumococcal diseases The Netherlands	IPD isolate	Validation set
2093933	22F	NRC for pneumococcal diseases The Netherlands	IPD isolate	Validation set
SSI 16	22F	NRC for pneumococcal diseases Denmark	IPD isolate	Validation set
SSI 36	22F	NRC for pneumococcal diseases Denmark	IPD isolate	Validation set
SSI 56	22F	NRC for pneumococcal diseases Denmark	IPD isolate	Validation set
SSI 76	22F	NRC for pneumococcal diseases Denmark	IPD isolate	Validation set
2200713	23A	NRC for pneumococcal diseases The Netherlands	IPD isolate	Validation set
SSI 20	23A	NRC for pneumococcal diseases Denmark	IPD isolate	Validation set
SSI 40	23A	NRC for pneumococcal diseases Denmark	IPD isolate	Validation set
SSI 60	23A	NRC for pneumococcal diseases Denmark	IPD isolate	Validation set
SSI 79	23A	NRC for pneumococcal diseases Denmark	IPD isolate	Validation set
SSI 21	23B	NRC for pneumococcal diseases Denmark	IPD isolate	Validation set
SSI 41	23B	NRC for pneumococcal diseases Denmark	IPD isolate	Validation set
SSI 61	23B	NRC for pneumococcal diseases Denmark	IPD isolate	Validation set
SSI 80	23B	NRC for pneumococcal diseases Denmark	IPD isolate	Validation set
200340	23F	NRC for pneumococcal diseases Belgium	IPD isolate	Validation set
2191285	23F	NRC for pneumococcal diseases The Netherlands	IPD isolate	Validation set
2202066	23F	NRC for pneumococcal diseases The Netherlands	IPD isolate	Validation set
2210058	23F	NRC for pneumococcal diseases The Netherlands	IPD isolate	Validation set
171439	24A	NRC for pneumococcal diseases Belgium	IPD isolate	Validation set
2111389	24B	NRC for pneumococcal diseases The Netherlands	IPD isolate	Validation set
SSI 93	24B	NRC for pneumococcal diseases Denmark	IPD isolate	Validation set
SSI 22	24F	NRC for pneumococcal diseases Denmark	IPD isolate	Validation set
SSI 42	24F	NRC for pneumococcal diseases Denmark	IPD isolate	Validation set
SSI 62	24F	NRC for pneumococcal diseases Denmark	IPD isolate	Validation set
SSI 81	24F	NRC for pneumococcal diseases Denmark	IPD isolate	Validation set
2080304	25A	NRC for pneumococcal diseases The Netherlands	IPD isolate	Validation set
2051283	25F	NRC for pneumococcal diseases The Netherlands	IPD isolate	Validation set

2050252	28A	NRC for pneumococcal diseases The Netherlands	IPD isolate	Validation set
2131817	28F	NRC for pneumococcal diseases The Netherlands	IPD isolate	Validation set
940744	32A	NRC for pneumococcal diseases The Netherlands	IPD isolate	Validation set
2081270	32F	NRC for pneumococcal diseases The Netherlands	IPD isolate	Validation set
2030777	33A	NRC for pneumococcal diseases The Netherlands	IPD isolate	Validation set
2080958	33A	NRC for pneumococcal diseases The Netherlands	IPD isolate	Validation set
2161448	33A	NRC for pneumococcal diseases The Netherlands	IPD isolate	Validation set
2180221	33A	NRC for pneumococcal diseases The Netherlands	IPD isolate	Validation set
840185	33B	NRC for pneumococcal diseases The Netherlands	IPD isolate	Validation set
2070895	33C	NRC for pneumococcal diseases The Netherlands	IPD isolate	Validation set
2070896	33D	NRC for pneumococcal diseases The Netherlands	IPD isolate	Validation set
SSI 17	33F	NRC for pneumococcal diseases Denmark	IPD isolate	Validation set
SSI 37	33F	NRC for pneumococcal diseases Denmark	IPD isolate	Validation set
SSI 57	33F	NRC for pneumococcal diseases Denmark	IPD isolate	Validation set
SSI 77	33F	NRC for pneumococcal diseases Denmark	IPD isolate	Validation set
2040179	35A	NRC for pneumococcal diseases The Netherlands	IPD isolate	Validation set
SSI 44	35B	NRC for pneumococcal diseases Denmark	IPD isolate	Validation set
SSI 64	35B	NRC for pneumococcal diseases Denmark	IPD isolate	Validation set
SSI 83	35B	NRC for pneumococcal diseases Denmark	IPD isolate	Validation set
SSI 88	35B	NRC for pneumococcal diseases Denmark	IPD isolate	Validation set
2040812	35C	NRC for pneumococcal diseases The Netherlands	IPD isolate	Validation set
SP12_D08	35D	Sciensano	NIPD isolate	Validation set
SP13_D01-M1	35D	Sciensano	NIPD isolate	Validation set
SP14_C03-M3	35D	Sciensano	NIPD isolate	Validation set
SSI 24	35D	NRC for pneumococcal diseases Denmark	IPD isolate	Validation set
2201923	35F	NRC for pneumococcal diseases The Netherlands	IPD isolate	Validation set
SP06_C40	35F	Sciensano	NIPD isolate	Validation set
SP08_C10	35F	Sciensano	NIPD isolate	Validation set
SP10_I08	35F	Sciensano	NIPD isolate	Validation set

SP13_G08	35F	Sciensano	NIPD isolate	Validation set
2081271	41A	NRC for pneumococcal diseases The Netherlands	IPD isolate	Validation set
2071854	41F	NRC for pneumococcal diseases The Netherlands	IPD isolate	Validation set
2081272	47A	NRC for pneumococcal diseases The Netherlands	IPD isolate	Validation set
2071858	47F	NRC for pneumococcal diseases The Netherlands	IPD isolate	Validation set
200166	6A	NRC for pneumococcal diseases Belgium	IPD isolate	Validation set
2200793	6A	NRC for pneumococcal diseases The Netherlands	IPD isolate	Validation set
2202252	6A	NRC for pneumococcal diseases The Netherlands	IPD isolate	Validation set
2210037	6A	NRC for pneumococcal diseases The Netherlands	IPD isolate	Validation set
SSI 91	6A	NRC for pneumococcal diseases Denmark	IPD isolate	Validation set
2200500	6B	NRC for pneumococcal diseases The Netherlands	IPD isolate	Validation set
2201713	6B	NRC for pneumococcal diseases The Netherlands	IPD isolate	Validation set
2202208	6B	NRC for pneumococcal diseases The Netherlands	IPD isolate	Validation set
2202213	6B	NRC for pneumococcal diseases The Netherlands	IPD isolate	Validation set
SSI 18	6C	NRC for pneumococcal diseases Denmark	IPD isolate	Validation set
SSI 38	6C	NRC for pneumococcal diseases Denmark	IPD isolate	Validation set
SSI 58	6C	NRC for pneumococcal diseases Denmark	IPD isolate	Validation set
191587	6D	NRC for pneumococcal diseases Belgium	IPD isolate	Validation set
2142139	6D	NRC for pneumococcal diseases The Netherlands	IPD isolate	Validation set
200314	7A	NRC for pneumococcal diseases Belgium	IPD isolate	Validation set
820999	7A	NRC for pneumococcal diseases The Netherlands	IPD isolate	Validation set
2110619	7A	NRC for pneumococcal diseases The Netherlands	IPD isolate	Validation set
2200021	7B	NRC for pneumococcal diseases The Netherlands	IPD isolate	Validation set
2200218	7C	NRC for pneumococcal diseases The Netherlands	IPD isolate	Validation set
SP05_E16	7C	Sciensano	NIPD isolate	Validation set
200174	7F	NRC for pneumococcal diseases Belgium	IPD isolate	Validation set
2200966	7F	NRC for pneumococcal diseases The Netherlands	IPD isolate	Validation set
2201172	7F	NRC for pneumococcal diseases The Netherlands	IPD isolate	Validation set
2201331	7F	NRC for pneumococcal diseases The Netherlands	IPD isolate	Validation set

2202201	7F	NRC for pneumococcal diseases The Netherlands	IPD isolate	Validation set
SSI 2	7F	NRC for pneumococcal diseases Denmark	IPD isolate	Validation set
SSI 94	7F	NRC for pneumococcal diseases Denmark	IPD isolate	Validation set
2000899	9A	NRC for pneumococcal diseases The Netherlands	IPD isolate	Validation set
2020010	9A	NRC for pneumococcal diseases The Netherlands	IPD isolate	Validation set
2120978	9A	NRC for pneumococcal diseases The Netherlands	IPD isolate	Validation set
2142048	9A	NRC for pneumococcal diseases The Netherlands	IPD isolate	Validation set
2160052	9A	NRC for pneumococcal diseases The Netherlands	IPD isolate	Validation set
2040413	9L	NRC for pneumococcal diseases The Netherlands	IPD isolate	Validation set
2051574	9L	NRC for pneumococcal diseases The Netherlands	IPD isolate	Validation set
190460	9N	NRC for pneumococcal diseases Belgium	IPD isolate	Validation set
190510	9N	NRC for pneumococcal diseases Belgium	IPD isolate	Validation set
SSI 28	9N	NRC for pneumococcal diseases Denmark	IPD isolate	Validation set
SSI 4	9N	NRC for pneumococcal diseases Denmark	IPD isolate	Validation set
SSI 48	9N	NRC for pneumococcal diseases Denmark	IPD isolate	Validation set
SSI 68	9N	NRC for pneumococcal diseases Denmark	IPD isolate	Validation set
SSI 86	9N	NRC for pneumococcal diseases Denmark	IPD isolate	Validation set
SSI 95	9N	NRC for pneumococcal diseases Denmark	IPD isolate	Validation set
181243	9V	NRC for pneumococcal diseases Belgium	IPD isolate	Validation set
2190431	9V	NRC for pneumococcal diseases The Netherlands	IPD isolate	Validation set
2190864	9V	NRC for pneumococcal diseases The Netherlands	IPD isolate	Validation set
2200496	9V	NRC for pneumococcal diseases The Netherlands	IPD isolate	Validation set
SSI 5	9V	NRC for pneumococcal diseases Denmark	IPD isolate	Validation set
SSI 85	9V	NRC for pneumococcal diseases Denmark	IPD isolate	Validation set
UKH_0170	9V	University Hospital Heidelberg	Not available	Testing influence of medium supplier
UKH_0356	7F	University Hospital Heidelberg	Not available	Testing influence of medium supplier
UKH_0381	9N	University Hospital Heidelberg	Not available	Testing influence of medium supplier
UKH_0403	6A	University Hospital Heidelberg	Not available	Testing influence of medium supplier
UKH_0502	5	University Hospital Heidelberg	Not available	Testing influence of medium supplier

UKH_0539	4	University Hospital Heidelberg	Not available	Testing influence of medium supplier
UKH_0556	18C	University Hospital Heidelberg	Not available	Testing influence of medium supplier
UKH_IB2770	3	University Hospital Heidelberg	Not available	Testing influence of medium supplier

Supplementary Table 2: List of all SG6 training set strains with their WGS serotype. The red shaded strain was initially typed as serotype 6A but after WGS and repeating the Quellung reaction, the strain was found to be serotype 6C. The blue shaded strain was phenotypically 6B (using both Quellung and FT-IR) but was found to be 6C based on WGS and we have currently no explanation for this discordance.

SG6 strain	Quellung serotype	WGS serotype	Quellung serotype repeated
191399	6A	6A	
191417	6A	6A	
2180226	6A	6A	
2193076	6A	6A	
2201720	6A	6A	
SP12_G05	6A	6C	6C
SP12_I38	6A	6A	
SP13_M04	6A	6A	
190154	6B	6B	
190736	6B	6B	
180659	6B	6B	
160886	6B	6B	
2193159	6B	6B	
SP12_I28	6B	6C	6B
200198	6C	6C	
200200	6C	6C	
5DCC053-03	6C	6C	
2180284	6C	6C	
2201527	6C	6C	
SP13_A39	6C	6C	
SP13_B21	6C	6C	
SP13_E17	6C	6C	
SP14_J35	6C	6C	

Supplementary Table 3: List of the validation set strains used, representing the 34 serotypes present in the training set. The red shaded serotypes each include one strain that is mistyped as NTS serotype using FT-IR.

	**Database Serotype**	**# of strains**
	**9N**	8
	**7F**	7
	**19F**	7
	**14**	6
	**18C**	6
	**4**	6
	**9V**	6
	**19A**	5
	**23A**	5
	**6A**	5
	**2**	5
	**22F**	5
	**38**	4
	**3**	4
	**8**	4
	**10A**	4
	**11A**	4
	**15A**	4
	**15B**	4
	**20**	4
	**23B**	4
	**24F**	4
	**16F**	4
	**12F**	4
	**6B**	4
	**23F**	4
	**5**	4
	**1**	4
	**17F**	4
	**35B**	4
	**33F**	4
	**6C**	3
	**24B**	2
	**24A**	1
**Total number**	**34 serotypes**	**153 strains**

Supplementary Table 4: List of the validation set strains used, representing 59 NTS serotypes. The numbers between brackets show extra strains that were measured after the initial analysis. The red shaded serotypes are mistyped as training set serotypes using FT-IR.

NTS Serotype	# of strains
15C	7
34	6
31	5
35F	5
21	4
35D	4
17A	3
13	3
7C	2
6D	2
9L	1 (+1)
27	1
29	1 (+3)
36	1
37	1
39	1 (+2)
40	1 (+4)
42	1
43	1
44	1
45	1
46	1
48	1
10B	1
10C	1
10F	1
11B	1
11C	1
11D	1 (+4)

NTS Serotype	# of strains
11F	1
12A	1
12B	1 (+1)
15F	1 (+2)
16A	1
18A	1 (+5)
18B	1
18F	1
19B	1 (+3)
19C	1
22A	1
25A	1
25F	1
28A	1
28F	1
32A	1
32F	1
33A	1 (+3)
33B	1
33C	1
33D	1
35A	1
35C	1
41A	1
41F	1
47A	1
47F	1
7A	1 (+2)
7B	1
9A	1 (+4)

Total number	59 serotypes	124 strains
